# Estimating Successful Internal Mobility: A Comparison Between Structural Equation Models and Machine Learning Algorithms

**DOI:** 10.3389/frai.2022.848015

**Published:** 2022-03-25

**Authors:** Francesco Bossi, Francesco Di Gruttola, Antonio Mastrogiorgio, Sonia D'Arcangelo, Nicola Lattanzi, Andrea P. Malizia, Emiliano Ricciardi

**Affiliations:** ^1^MoMiLab Research Unit, IMT School for Advanced Studies Lucca, Lucca, Italy; ^2^Axes Research Unit, IMT School for Advanced Studies Lucca, Lucca, Italy; ^3^Neuroscience Lab, Intesa Sanpaolo Innovation Center SpA, Turin, Italy

**Keywords:** internal mobility, job relocation, job satisfaction, structural equation models, machine learning, resistance to change, predictive HR analytics

## Abstract

Internal mobility often depends on predicting future job satisfaction, for such employees subject to internal mobility programs. In this study, we compared the predictive power of different classes of models, i.e., (i) traditional Structural Equation Modeling (SEM), with two families of Machine Learning algorithms: (ii) regressors, specifically least absolute shrinkage and selection operator (Lasso) for feature selection and (iii) classifiers, specifically Bagging meta-model with the *k*-nearest neighbors algorithm (*k*-NN) as a base estimator. Our aim is to investigate which method better predicts job satisfaction for 348 employees (with operational duties) and 35 supervisors in the training set, and 79 employees in the test set, all subject to internal mobility programs in a large Italian banking group. Results showed average predictive power for SEM and Bagging *k*-NN (accuracy between 61 and 66%; F1 scores between 0.51 and 0.73). Both SEM and Lasso algorithms highlighted the predictive power of resistance to change and orientation to relation in all models, together with other personality and motivation variables in different models. Theoretical implications are discussed for using these variables in predicting successful job relocation in internal mobility programs. Moreover, these results showed how crucial it is to compare methods coming from different research traditions in predictive Human Resources analytics.

## Introduction

Job relocation is a traditional issue of organizational literature whose main paradigms refer to the effect of job transfer on stress and family life (Burke, 1986; Munton, 1990), where job transfer traditionally requires geographical mobility. Consolidate evidence shows that the preference for a specific location is a major predictor of post-transfer satisfaction (Pinder, 1977). In general, employees in the early career stage tend to be more willing to accept mobility opportunities as they perceive more dissonance between their current job and ideal job (Noe et al., 1988). The willingness to relocate enters the selection process in which attitudinal, biographical and social variables predict how many potential employees are prone to international mobility (Andresen and Margenfeld, 2015).

Nevertheless, post-transfer satisfaction is not simply a matter of geographical opportunity. The rise of information technologies in recent decades has made geographical relocations less problematic, as they enable a flexible and geographically independent job organization (i.e. remote working). Internal migration rates are declining across most Western countries (Haan and Cardoso, 2020), for several economic and social reasons. In contemporary economies, post-transfer satisfaction is mainly referred to the *internal mobility* where the changes—due to promotions and/or lateral transfers—occur within the same organization. Promoted workers, internal to an organization, have significantly better performance and lower exit rates than those externally hired into similar jobs (Bidwell, 2011). Indeed, upward progressions are much more likely to happen through internal than external mobility (Bidwell and Mollick, 2015). High performers are less likely to quit, and when they do quit the reasons are typically not related to work (Benson and Rissing, 2020). Furthermore, there is evidence of a negative association between performance and internal mobility for low performers as they add value to the organization by developing complex social networks through internal job transfers (Chen et al., 2020a).

Internal mobility is not just an opportunity for career development. In many cases, internal mobility is not a discretional choice but a strategic or a contingent organizational need that could involve the forced relocation of hundreds of employees. In such cases, *predicting job satisfaction* for such employees involved in mobility programs is fundamental. The literature on job satisfaction is abundant regarding the construct and its antecedents (e.g., Judge et al., 2002; Aziri, 2011), but its prediction is often problematic. In particular, job satisfaction is not always an existent construct to be simply measured in given settings. Human resources (HR) specialists are often interested in predicting post-transfer job satisfaction in such settings that include internal mobility, as organizational changes are designed precisely depending on how employees will react to the new arrangements. In short, internal mobility often depends on the prediction of future job satisfaction. In such situations, what HR practitioners have at disposal is many individual-related variables, such as individual differences in personality, motivation and emotion for workers, and leadership style and empathy for leaders. Using such variables to predict job satisfaction—where satisfaction is a general construct that also includes communication- and inclusion-related aspects—could be opportune. Machine learning comes in help as it allows predicting job satisfaction, based on the available variables.

### Job Satisfaction

Job satisfaction represents a complex research domain stratified over decades, whose definition and research questions are significantly dependent on the specific historical contingencies (Latham and Budworth, 2007). Generally speaking, job satisfaction is a construct whose investigation admits different paradigms and approaches, each one with specific theoretical nuances. Such approaches include Hertzberg's motivator-hygiene theory (Herzberg, 1964), job design frameworks (Hackman and Oldham, 1976), dispositional (Staw et al., 1986) and equity approaches (Huseman et al., 1987). Traditionally, job satisfaction has been defined as “a pleasurable or positive emotional state resulting from the appraisal of one's job or job experiences” (Locke, 1976, p. 1304). Job satisfaction presents a number of facets as it can be defined with reference to specific job aspects. Spector (1997) identifies fourteen aspects that include appreciation, communication, coworkers, fringe benefits, job conditions, nature of the work, organization, personal growth, policies and procedures, promotion opportunities, recognition, security, and supervision.

The assumption that happier workers are more productive is the fundamental hypothesis of literature, showing that both cognitive and affective factors can explain, to different degrees, job satisfaction (Moorman, 1993). Managers usually look for satisfied workers, assuming that they are more engaged and performative, where job satisfaction and employee motivation, though different constructs, are fundamental for organizational performance (Vroom, 1964). The meta-analytical evidence of satisfaction-performance relationship encompasses several paradigms that flourished over the last century, whose theoretical and practical implications would deserve dedicated discussions (Schwab and Cummings, 1970; Iaffaldano and Muchinsky, 1985; Judge et al., 2001; Harter et al., 2002). Importantly, job satisfaction traditionally also extends outside of the job domains to include private life (Near et al., 1980; Rain et al., 1991). The “happy-productive worker paradigm” has been unpacked and evidence shows the role of general psychological well-being, not just job satisfaction, in explaining performance (Wright and Cropanzano, 2000). While such meta-analytical evidence emphasizes a correlation between job satisfaction and individual performance, the same cannot be maintained for organizational performance, where the less consolidated literature shows mixed evidence. Some studies show a positive relationship (e.g., Huselid, 1995; Schneider et al., 2003), others show the absence of any significant correlation (e.g., Mohr and Puck, 2007). Interestingly, the opposite relationship is also meaningful considering that organizational success affects employees' satisfaction (Ryan et al., 1996).

### Predictive HR Analytics

Big data analytics represent a fundamental factor for companies to mine information to achieve competitive advantages (for a generalist literature review see Holsapple et al., 2014; Chong and Shi, 2015). Within this broad domain, HR analytics occupies a significant position as they help companies in managing human resources by exploiting data about how employees work and their individual differences. HR analytics refers to the use of statistical tools and computational methods for making decisions involving HR strategies and practices.

While HR analytics are traditionally reactive, predictive HR analytics is proactive and represents a relatively novel domain of investigation. Predictive HR analytics can be defined as “the systematic application of predictive modeling using inferential statistics to existing HR people-related data to inform judgements about possible causal factors driving key HR-related performance indicators” (Edwards and Edwards, 2019, p. 3). The increasing application of artificial intelligence (i.e., machine learning), far from being a passing fad, represents a significant trend in the last decade (Falletta, 2014). Predictive HR analytics serve the purpose of identifying opportunities and risks in advance before they are clear to managers. Finally, predictive HR analytics is not merely devoted to improving efficiency but, more and more enables strategic human capital decisions (Kapoor and Sherif, 2012; Zang and Ye, 2015).

HR analytics is a still developing topic whose related evidence is often based on anecdotal evidence and case histories (e.g., Dow Chemical mined the employee data to predict the success of promotions and internal transfers, Davenport et al., 2010). Ben-Gal (2019), through an analytical review of the literature, highlights that empirical and conceptual studies in HR analytics are related to higher economic performances compared to technical- and case-based studies. In particular, such performances are related to the application of HR analytics to workforce planning and recruitment/selection tasks.

While the general impact of artificial intelligence on HR is a well-debated topic (Bassi, 2011; e.g., Rathi, 2018), the study of specific machine learning methods for predictive purposes in HR analytics represents a non-consolidated domain of research, characterized by a high degree of technicalities (Kakulapati et al., 2020). Such research domain includes the turnover prediction through neural networks (Quinn et al., 2002) or machine learning algorithms based on Extreme Gradient Boosting (Punnoose and Ajit, 2016), data mining for personnel selection (Chien and Chen, 2008), workforce optimization through constraint programming (Naveh et al., 2007). Nevertheless, in the past decades, the application of automated machine learning algorithms or neural networks in this field was mostly limited to the areas of intervention of a company spread in a region (Kolesar and Walker, 1974) or to the relocation of a whole company to a new geographical location (Haddad and Sanders, 2020). However, no studies have previously compared different statistical methods for predictive HR analytics or, more specifically, for automated relocation.

A crucial problem of predictive HR analytics is related to ethical issues arising from evidence-based decisions. Indeed, the use of some specific predictive variables can be problematic: what if HR specialists make human capital decisions based, e.g., on an applicant's hometown, car preference or sports habits, precisely because these variables are predictive of job performance? Such practices might be questionable and represent a matter on which the HR community will be likely called into account in the years to come (for a discussion see, Hamilton and Davison, 2021). In particular, some of the main concerns include the violation of national and international employment discrimination laws or data protection regulations, as well as employees' desires for privacy and justice.

### Aim of the Study

In this study, we compared the predictive power of three classes of models. We compared (i) traditional Structural Equation Modeling (SEM), with two families of Machine Learning algorithms, i.e., (ii) regressors, specifically least absolute shrinkage and selection operator (Lasso) for feature selection and (iii) classifiers, specifically Bootstrap Aggregation meta-model using as base estimator the *k*-nearest neighbors algorithm (*k*-NN). Our aim is to validate which method better predicts successful relocation (measured in terms of job satisfaction, inclusion in the work team, and communication satisfaction) for 348 employees (with operational duties) and 35 leaders in the training set, and 79 employees in the test set, subject to internal mobility programs in a large Italian banking group. We considered an array of heterogeneous independent variables (from personality and motivation literature) which often constitute what HR directors have at disposal for making predictions about their employees, and we compared the alternative predictive methods.

## Materials and Methods

### Participants

During the first part of the study (training set, February-March 2020), 503 employees and 40 supervisors in a large-scale Italian banking group volunteered for the data collection. Out of this sample, 380 employees and 37 supervisors opened the survey, but only 348 employees (147 F, mean ± sd age: 49.4 ± 6.9) and 35 supervisors (7 F, mean ± sd age: 50.7 ± 7.5) completed the survey. During the second data collection (test set, July 2020), 100 employees volunteered, 82 of them opened the survey and 79 (34 F, mean ± sd age: 48.3 ± 7.6) completed it. All employees were relocated more than 6 months before the data collection (mean training set: 15.9 months; mean test set: 14.1 months). All participants had normal or a corrected-to-normal vision, no history of auditory or psychiatric disorders.

#### Ethical Statement

All participants were provided with an exhaustive description of all the experimental procedures and were required to sign a written informed consent before taking part in the study. The study was conducted in accordance with the ethical standards laid down in the 1964 Declaration of Helsinki and under a protocol approved by the Area Vasta Nord Ovest Ethics Committee (protocol n. 24579/2018).

### Procedure

All questionnaires were administered via an online survey based on SurveyMonkey®. Survey links were sent by email to all volunteers by a collaborator bank employee. In this way, researchers could never have direct access to participants' names and they could participate anonymously. The first part of data collection (348 employees and 35 supervisors), aimed to collect the training set, was carried out between February 26th and March 16th 2020. The second part of data collection (79 employees), aimed to collect the test set, was carried out between July 6th and July 31st 2020.

### Materials

Questionnaires administered to employees (in both training and test sets) included a Personality questionnaire (Jackson et al., 1996a,b; Hogan and Hogan, 1997, 2007), Motivational Orientation Test (Alessandri and Russo, 2011), Resistance to Change questionnaire (Oreg, 2003), Emotion Regulation Questionnaire (Gross and John, 2003), Rational Experiential Inventory – Short Form (REI-S24, Pacini and Epstein, 1999), Inclusion questionnaire (Jansen et al., 2014), Job Satisfaction Index (Brayfield and Rothe, 1951), and Communication Satisfaction Scale (Madlock, 2008). Questionnaires administered to supervisors (only in the training set) included the Trait Emotional Intelligence Questionnaire – Short Form (Petrides, 2009), Interpersonal Reactivity Instrument (Davis, 1983), Prosocialness Scale for Adults (Caprara et al., 2005), Multifactor Leadership Questionnaire – 6S Form (Avolio and Bass, 2004), and REI-S24 (Pacini and Epstein, 1999).

#### Predictors

Questionnaires used to measure predictors (or independent variables) administered to employees are:

##### Personality Questionnaire

We built a 40-items questionnaire (Malizia et al., 2021) to measure four specific dimensions of personality of interest in our study. In particular, we considered three dimensions of the Six Factor Personality Questionnaire (Jackson et al., 1996a,b). Such dimensions (and their facets) are Independence (autonomy, individualism, self-reliance), Openness to Experience (change, understanding, breadth of interest), Industriousness (achievement, endurance, seriousness). A further dimension, Dutifulness, was selected from the Hogan Personality Inventory (Hogan and Hogan, 1997, 2007).

##### Motivational Orientation Test

The Motivational Orientation Test (Borgogni et al., 2004; Petitta et al., 2005; Test di Orientamento Motivazionale, see Alessandri and Russo, 2011) is based on 43 items and addressed to measure four drivers—Objective, Innovation, Relation, Leadership—of individual motivation.

##### Resistance to Change

The Resistance to Change Test (Oreg, 2003), based on 18 items, was used to measure four dimensions related to change: Routine Seeking, Emotional Reaction to Imposed Change, Cognitive Rigidity, and Short-Term Focus. We used the total index in our analyses as it showed higher reliability in the original validation paper.

##### Emotion Regulation

Participants' use of different emotion regulation strategies was investigated with the Emotion Regulation Questionnaire (ERQ, Balzarotti et al., 2010). This is a 10-item questionnaire, in which each item is scored on a 7-point Likert scale (from 1 = “Strongly disagree” to 7 = “Strongly agree”). Items are scored into two separate subscales investigating expressive suppression (basic emotion regulation strategy, i.e., suppressing the behavioral expression of the emotion) and cognitive reappraisal (more advanced cognitive emotion regulation strategy, aimed at modifying the internal representation of an event to change one's own emotional experience) (Gross and John, 2003). Previous literature (*ibidem*) showed that people who use cognitive reappraisal more often tend to experience and express greater positive emotion and lesser negative emotion, whereas people who use expressive suppression experience and express lesser positive emotion, yet experience greater negative emotion.

Questionnaires used to measure predictors (or independent variables) administered to supervisors are:

##### Trait Emotional Intelligence Questionnaire—Short Form

The Trait Emotional Intelligence Questionnaire—Short Form (Petrides, 2009), based on 30 items, was used to measure trait emotional intelligence.

##### Interpersonal Reactivity Instrument

The Interpersonal Reactivity Instrument (Davis, 1983) based on 28 items, is aimed at measuring dispositional empathy on four dimensions: Perspective Taking, Fantasy, Empathic Concern and Personal Distress.

##### Prosocialness Scale for Adults

The Prosocialness Scale for Adults (Caprara et al., 2005), based on 16 items, was used to measure individual differences in adult prosocialness.

##### Multifactor Leadership Questionnaire

The shortened form of the Multifactor Leadership Questionnaire (Avolio and Bass, 2004), based on 21 items, was used to measure transformational and transactional leadership.

The only questionnaire used to measure predictors (or independent variables) administered to both groups is:

##### Rational Experiential Inventory–Short Form (REI-S24)

We used the Rational Experiential Inventory (Pacini and Epstein, 1999), in the short version of 24 items, to measure to what degree people engage in automatic-System 1 or deliberate-System 2 modes of thinking.

#### Outcomes

Questionnaires used to measure outcomes (or dependent variables) administered to employees are:

##### Inclusion Questionnaire

The Perceived Group Inclusion Scale (Jansen et al., 2014), composed of 16 items, was used in order to measure inclusion in the workplace.

##### Job Satisfaction Index

We used the traditional index of job satisfaction (Brayfield and Rothe, 1951), based on 18 items.

##### Communication Satisfaction Scale

The 19-items Communication Satisfaction Scale (adapted from Madlock, 2008) was used to understand the influence of supervisor communication competence on employees satisfaction.

### General Data Treatment

All questionnaires were scored according to official guidelines from each validation paper (which typically consisted of summing scores from all items in each factor). Since it was impossible to trace back the exact correspondence between each colleague and individual supervisors for privacy reasons, average scores were computed for supervisors in each of the six business units involved in the project. These scores were then attributed to each colleague according to their business unit when computing models and algorithms.

In all approaches, the Inclusion questionnaire, Job Satisfaction Index, and Communication Satisfaction Scale were considered as three outcomes of success in job relocation (i.e., endogenous variables in Structural Equation Models and target measures in machine learning algorithms). Accordingly, all other variables from employees (Independence, Openness, Industriousness, Dutifulness, Orientation to Target, to Innovation, to Relation, to Leadership, Resistance to Change, Cognitive Reappraisal, Expressive Suppression, Rational Style, Experiential Style, Age, Seniority) and supervisors (Emotional Intelligence, Empathy, Prosociality, six Leadership Styles, Rational Style, Experiential Style, Age, Seniority) were used as predictors (i.e., exogenous variables in SEM and features in machine learning algorithms).

The Mean Absolute Error (MAE) was used in both SEM and machine learning algorithms to compare the prediction accuracy in the test set. The MAE is a common measure of prediction accuracy in regression models and is computed according to Formula 1:


(1) $$\begin{array}{r} MAE=\frac{\overset{n}{\underset{t=1}{\sum }}|P_{t}-O_{t}|}{n} \end{array}$$

where *O*_*t*_ is the observed value and *P*_*t*_ is the predicted value. The absolute value in their difference is summed for every predicted point and divided by the number of fitted points *n*.

Data collected from participants involved in the first data collection (i.e., 348 employees and 35 supervisors) were used as a training set; data collected from participants involved in the second data collection (i.e., 79 employees) were used as an independent test set.

### Structural Equation Models

When using the Structural Equation Models (SEM) approach, the analyses were aimed to find the most efficient model in predicting success in job relocation, i.e., predicting the highest variance with the lower number of parameters. Given this aim, the most suitable method to compare models is the Akaike Information Criterion (AIC, Akaike, 1974). The AIC is a goodness of fit index and therefore evaluates how well a model fits the data it was generated from. Let *k* be the number of estimated parameters in the model and let $\hat{L}$ be the maximum value of the likelihood function for the model: as shown in Formula 2, the AIC also takes into account the model complexity, as it is penalized for the number of parameters included in the model. This penalty is aimed at reducing overfitting. When comparing different models, the model with the lowest AIC is also the most efficient one (i.e., explaining more variance with fewer parameters).


(2) $$\begin{array}{r} AIC=2k-ln\left(\hat{L}\right) \end{array}$$

In the models comparison procedure, we started by testing models with the highest number of exogenous variables and then reducing the parameters estimated by the model by removing parameters that did not show a statistically significant effect on the endogenous variables. The whole models comparison procedure is detailed in the results. In all models, the estimator was Maximum Likelihood (ML) and the optimization method was Nonlinear Minimization subject to Box Constraints (NLMINB).

Fit measures (i.e., Comparative Fit Index (CFI), Tucker-Lewis Index (TLI), Root Mean Square Error of Approximation (RMSEA), Standardized Root Mean Square Residual (SRMR), Akaike Information Criterion (AIC) and R^2^) from all models are reported in Table 1. We indicate that R^2^ is a goodness of fit measure, as it shows the explained variance of the outcome variable predicted by the predictors (ranging from 0 to 1), but it is not penalized for the number of parameters in the model as the AIC.

**Table 1:** Structural Equation Models summary.

	**M1**	**M2**	**M3**	**M4**
Number of free parameters	60	57	45	17
Comparative Fit Index (CFI)	> 0.999	> 0.999	> 0.999	0.992
Tucker-Lewis Index (TLI)	> 0.999	> 0.999	> 0.999	0.962
Root Mean Square Error of Approximation (RMSEA)	<0.001	<0.001	<0.001	0.049
Standardized Root Mean Square Residual (SRMR)	<0.001	<0.001	<0.001	0.027
Akaike Information Criterion (AIC)	8,123	8,130	8,113	8,083
R-Square:				
INQ_Inclusion	0.173	0.169	0.159	0.138
JSI_JobSatisfaction	0.185	0.162	0.155	0.129
CSS_CommunicationSatisfaction	0.176	0.175	0.169	0.137

*Acronyms for all tables: INQ, Inclusion Questionnaire; JSI, Job Satisfaction Index; CSS, Communication Satisfaction Scale; PQ, Personality Questionnaire; TOM, Motivational Orientation Test (original name: Test di Orientamento Motivazionale); ERQ, Emotion Regulation Questionnaire; REIS24, Rational-Experiential Inventory—Short form 24 items; TEIQ, Trait Emotional Intelligence Questionnaire; IRI, Interpersonal Reactivity Instrument; PSA, Prosocialness Scale for Adults*.

Structural equation models were analyzed in RStudio software (RStudio Inc., 2016) by using the *lavaan* package (Rosseel, 2012).

### Machine Learning Algorithms

Through Machine Learning we aimed at selecting the best features to predict the target variables with a certain degree of accuracy. We opted for using a Least Absolute Shrinkage and Selection Operator (Lasso) regression algorithm for feature selection and a Bootstrap Aggregation of K-Nearest Neighbors classifiers for final classification. Despite having continuous target variables, the latter choice was made in order to reduce data variability given the small sample size. We used Python's Pandas (Version 1.3.3), Numpy (1.21.2) and Scikit-Learn (1.0) toolboxes for this analysis setting seed of 1.

Firstly, some further pre-processing steps were carried out on the data before the model validation process. For each feature, the score of the training set was normalized to obtain a distribution with mean 0 and standard deviation 1 (Z-scores transformation). The mean and standard deviation of the training set distribution were also used as a benchmark to normalize the data points of the test set features. Regarding the target variables, to apply the regression model, no further pre-processing steps were needed. On the other hand, to use the classification algorithm, we transformed each target variable of the training set into an ordinal dichotomous output through a median split, assigning labels of 1 and 2 to the values below and above the median, respectively. We used the same median value of the training set to split and transform the test set target variables.

Then, three types of Machine Learning models were used for the analysis: Lasso Regression, K-Nearest Neighbors Classifier and Bootstrap Aggregation meta-model. The latter is an ensemble learning model created with a bootstrapping method and used to further enhance the performance of a single K-Nearest Neighbors Classifier. For each target variable (Inclusion, Job Satisfaction and Communication Satisfaction), an independent model validation process was followed, thus obtaining three Lasso, three K-Nearest Neighbors and three Bootstrap Aggregation meta-models in total. We also evaluated different classification methods to be used instead of K-Nearest Neighbors; see Supplementary Material for a complete description of the classification method choice.

#### Lasso Regression

We used Lasso regression to select the best features for each model. This step can be useful when dealing with small datasets, as in our case, in order to reduce overfitting and the curse of dimensionality (Chen et al., 2020b). Lasso is a linear regression model where the absolute value of each feature coefficient is added to the loss function (Ordinary Least Square) and multiplied to a constant parameter Alpha (Friedman et al., 2010). This type of regularization (L1) allowed us to perform feature selection, zeroing the coefficient of the less important features in predicting the target variable and using only the remaining ones in the model. This feature made the Lasso Regression one of the three reference models in our research because has a similar objective and output of SEMs and is useful in reducing the numerous features we have in our small sample size dataset.

A Randomized Search Cross-Validation fitted on the training set was used to find parameters that optimize the Lasso regression model performance (hyperparameter tuning—Bergstra and Bengio, 2012). Considering the trade-off between search quality and computational efficiency, we set n= 100 randomized search iterations. For each loop, the algorithm assesses a certain number of random combinations by picking up from a starting grid an entry for each validation parameter. In our case, we made the Lasso hyperparameter tuning only on the Alpha parameter. Accordingly, to select the best Alpha for each model we used a grid of values ranging from 0.1 to 20 in steps of 0.1 (n = 200 maximum Alpha values to select). Then, for each iteration, the model performance is measured using a K-Fold Cross-Validation score (Géron, 2019). This technique avoids a fixed split of the data into a training and a validation set. Accordingly, the algorithm divides the training set into K parts, in our case K = 5 (as a trade-off between quality and computation timing), computing 5 iterations. For each K, one-fifth of the training set was in turn used as validation and the other part as the training set computing a performance score each time. The mean of the MAE for the 5-Fold iterations was the final cross-validation score of each randomized search iteration. Then, the Alpha value which led the model to the lowest MAE was picked and the best model was used for feature selection. For each feature, we obtained a regression coefficient computed on the training set that reflects feature importance in predicting the target variable. This coefficient could be positive or negative and can be interpreted as the increase or decrease, respectively, in the target variable score for one standard deviation change of the feature. For each target variable, features with a coefficient equal to zero have been discarded and not included in the validation process as starting predictors of the K-Nearest Neighbors Classifier.

#### K-Nearest Neighbors Classifier

The K-Nearest Neighbors was used as the base model for the Bootstrap Aggregation meta-model in order to solve the classification problem. This approach was chosen because it is one of the simplest machine learning classification models. In fact, for each data point, this model predicts the target label by looking at the K closest data points (Géron, 2019). For the Randomized Cross-Validation of the model, we tuned three parameters: *number of neighbors, weight* and *metric*. For the *number of neighbors*, thus the K closest point to consider for the classification, a grid was used with odd values ranging from 1 to 85 for both the Inclusion and the Communication Satisfaction target variables, while from 1 to 81 for the Job Satisfaction. This range was chosen in order to avoid overfitting because the maximum possible number of K was equal to half the data points belonging to the least represented class of each target variable. The *weight* parameter, that controls the importance assigned to the K neighbors, had two entries: *uniform*, which assigns the same weight to the neighbors, leading to choose the predicted label according to the most frequent and closest K and *distance* that gives proportionally high weight to the nearest points. *Metric* is the formula used to calculate the distance between data points. The entries are *Manhattan* and *Euclidean* that use an L1 and L2 norm formula, respectively. The model performance was assessed by the accuracy score–i.e., the number of correct predictions out of the total, the higher, the better. Finally, the optimized model was used on the test set. This operation was carried out with two purposes: to assess the goodness of the single K-Nearest Neighbors classifier, thus lower or better model performance for the training set compared to the test set, respectively; and as a control measure to understand whether the ensemble learning technique would actually lead to improvements to the single base model in terms of performance. This possibility was evaluated using again the accuracy score. Moreover, given the small sample size and the imbalance between classes, we also computed, for each target class, the *precision*, that is the number of correctly predicted samples of the class with respect to all the samples predicted of that class by the classifier, *recall*, which is the number of correctly classified samples of that class compared to the total samples of the same class and *F1 score*, that is a weighted average of precision and recall scores (Saito and Rehmsmeier, 2015).

#### Bagging Meta-Model

We used an ensemble meta-estimator called Bootstrap Aggregation (Bagging–Breiman, 1996) with each single validated K-Nearest Neighbors classification model. We chose this approach in order to reduce the variance of the single model, that is the error deriving from the noise present in the training sample, whose consequence could be overfitting. The Bagging allows us to train each validated model n times picking at random and with replacement for each iteration 348 data points, corresponding to the sample size of the training set. Thus, some data points may be picked more than once in the same iteration, while others may never be drawn (out of bagging). For each iteration, the trained model makes a prediction. For the classifier (Bagging Classifier) the final prediction of the target variable is the most frequent predicted class.

In the validation process of each bagging meta-model, we chose the best number of iterations with a for loop testing. We set a range of n going from 10 to 100 with a step of 1 as a trade-off between accuracy and computation power. For each iteration, a 5-Fold Cross-Validation on the training set was done by computing the mean and standard deviation of the accuracy score as the performance metric. The iteration parameter that reflected the model with the highest mean accuracy score was considered to be the best one. Then, an optimized meta-model was fitted on the training set and the model performance was evaluated on the test set with accuracy, precision, recall and F1 scores. Specifically, the accuracy was used to assess the possible presence of underfitting or overfitting. The latter conditions were operationalized as a variation of more than one standard deviation between the test set and the training set accuracy scores.

## Results

## Structural Equation Models

All model summaries can be found in Table 1, while statistically significant parameters are reported in Table 2. A report of all parameters in all models can be found in the Supplementary Material, in which statistically significant effects are highlighted in bold.

**Table 2:** Summary of statistically significant parameters.

**M1**						
**Regressions**						
	**Estimate**	**Std. Err**	***z*** **value**	**P (>|z|)**	**Std. lv**	**Std. all**
**INQ_Inclusion** **~**						
PQ_Industriousness	−0.422	0.182	−2.316	0.021	−0.422	−0.137
PQ_Dutifulness	0.439	0.173	2.534	0.011	0.439	0.149
TOM_Relation	0.577	0.176	3.271	0.001	0.577	0.220
Resistance to change	−0.170	0.077	−2.192	0.028	−0.170	−0.147
**JSI_JobSatisfaction** **~**						
TOM_Relation	0.560	0.191	2.929	0.003	0.560	0.196
Resistance to change	−0.287	0.084	−3.420	0.001	−0.287	−0.227
**CSS_CommunicationSatisfaction** **~**						
PQ_Industriousness	−0.410	0.194	−2.115	0.034	−0.410	−0.125
PQ_Dutifulness	0.429	0.184	2.327	0.020	0.429	0.137
TOM_Target	0.384	0.174	2.207	0.027	0.384	0.207
TOM_Relation	0.647	0.188	3.446	0.001	0.647	0.231
Resistance to change	−0.214	0.082	−2.597	0.009	−0.214	−0.173
**M2**						
**Regressions**						
**INQ_Inclusion** **~**						
PQ_Industriousness	−0.418	0.182	−2.300	0.021	−0.418	−0.136
PQ_Dutifulness	0.444	0.174	2.555	0.011	0.444	0.151
TOM_Relation	0.615	0.179	3.438	0.001	0.615	0.234
Resistance to change	−0.188	0.077	−2.448	0.014	−0.188	−0.162
**JSI_JobSatisfaction** **~**						
TOM_Relation	0.593	0.196	3.028	0.002	0.593	0.207
Resistance to change	−0.323	0.084	−3.846	<0.001	−0.323	−0.255
**CSS_CommunicationSatisfaction** **~**						
PQ_Industriousness	−0.413	0.193	−2.137	0.033	−0.413	−0.126
PQ_Dutifulness	0.439	0.185	2.378	0.017	0.439	0.140
TOM_Target	0.379	0.175	2.169	0.030	0.379	0.204
TOM_Relation	0.664	0.190	3.493	<0.001	0.664	0.237
Resistance to change	−0.212	0.081	−2.605	0.009	−0.212	−0.172
**M3**						
**Regressions**						
**INQ_Inclusion** **~**						
PQ_Industriousness	−0.368	0.180	−2.044	0.041	−0.368	−0.119
PQ_Dutifulness	0.421	0.174	2.422	0.015	0.421	0.143
TOM_Relation	0.598	0.177	3.369	0.001	0.598	0.228
Resistance to change	−0.187	0.077	−2.433	0.015	−0.187	−0.161
**JSI_JobSatisfaction** **~**						
TOM_Relation	0.606	0.194	3.120	0.002	0.606	0.211
Resistance to change	−0.320	0.084	−3.804	<0.001	−0.320	−0.253
**CSS_CommunicationSatisfaction** **~**						
PQ_Industriousness	−0.401	0.191	−2.099	0.036	−0.401	−0.122
PQ_Dutifulness	0.429	0.184	2.330	0.020	0.429	0.137
TOM_Target	0.384	0.174	2.202	0.028	0.384	0.206
TOM_Relation	0.652	0.188	3.466	0.001	0.652	0.233
Resistance to change	−0.208	0.081	−2.558	0.011	−0.208	−0.169
**M4**						
**Regressions**						
**INQ_Inclusion** **~**						
PQ_Industriousness	−0.299	0.141	−2.117	0.034	−0.299	−0.097
PQ_Dutifulness	0.292	0.133	2.193	0.028	0.292	0.099
TOM_Relation	0.793	0.136	5.827	<0.001	0.793	0.303
Resistance to change	−0.215	0.058	−3.708	<0.001	−0.215	−0.186
**JSI_JobSatisfaction** **~**						
TOM_Relation	0.755	0.143	5.269	<0.001	0.755	0.264
Resistance to change	−0.298	0.063	−4.712	<0.001	−0.298	−0.236
**CSS_CommunicationSatisfaction** **~**						
PQ_Dutifulness	0.348	0.148	2.351	0.019	0.348	0.112
TOM_Relation	0.755	0.155	4.864	<0.001	0.755	0.271
Resistance to change	−0.242	0.069	−3.493	<0.001	−0.242	−0.196

*Std. lv, effects estimate standardized on the first manifest variable; in our models, this corresponds to the default estimate. Std. all, effects estimate standardized on all manifest variables*.

The first model included all variables collected from both employees and supervisors (excluding age and seniority). Nevertheless, in this model, the sample covariance matrix was not positive-definite. This result typically implies multicollinearity in the model (i.e., means that at least one of the exogenous variables can be expressed as a linear combination of the others) or the number of observations is less than the number of variables. Best practice, in this case, is to remove highly correlated variables from the model (Field et al., 2012); in our case, the questionnaire showing the highest number of highly correlated variables (i.e., |r| > 0.5) was the Multifactor Leadership Questionnaire (MLQ-6S). For this reason, this model was re-run without the 6 Leadership Styles variables.

The following model (M1) included variables collected from both employees and supervisors (excluding Leadership Styles, age and seniority). Information and fit indices from this and the following models are summarized in Table 1. Since no variables collected from supervisors showed statistically significant effects on any of the three endogenous variables, we removed these exogenous variables and added age and seniority (from both employees and supervisors) to the next model (M2). Also in this case, age and seniority (from both employees and supervisors) showed no statistically significant effects on any of the three endogenous variables. Therefore, in M3 only variables collected from employees were included. This model was further reduced by including only parameters showing significant effects in M3 (in a feature selection fashion), leading to an optimized model (M4). As shown in Table 1, the AIC was lower in M4 (8083) than in M3 (8113), displaying thus increased efficiency in explaining data in the optimized model (i.e., M4) compared to M3. Nevertheless, a Likelihood Ratio Test (LRT) was performed between the two best models (i.e., M3 and M4) to compare the likelihood of the two models. This LRT showed that the models' likelihood was not significantly different (Δχ^2^ (4) = 7.35, *p* = 0.119), despite the relevant change in the number of estimated parameters (45 in M3 vs. 17 in M4).

**Figure 1:**
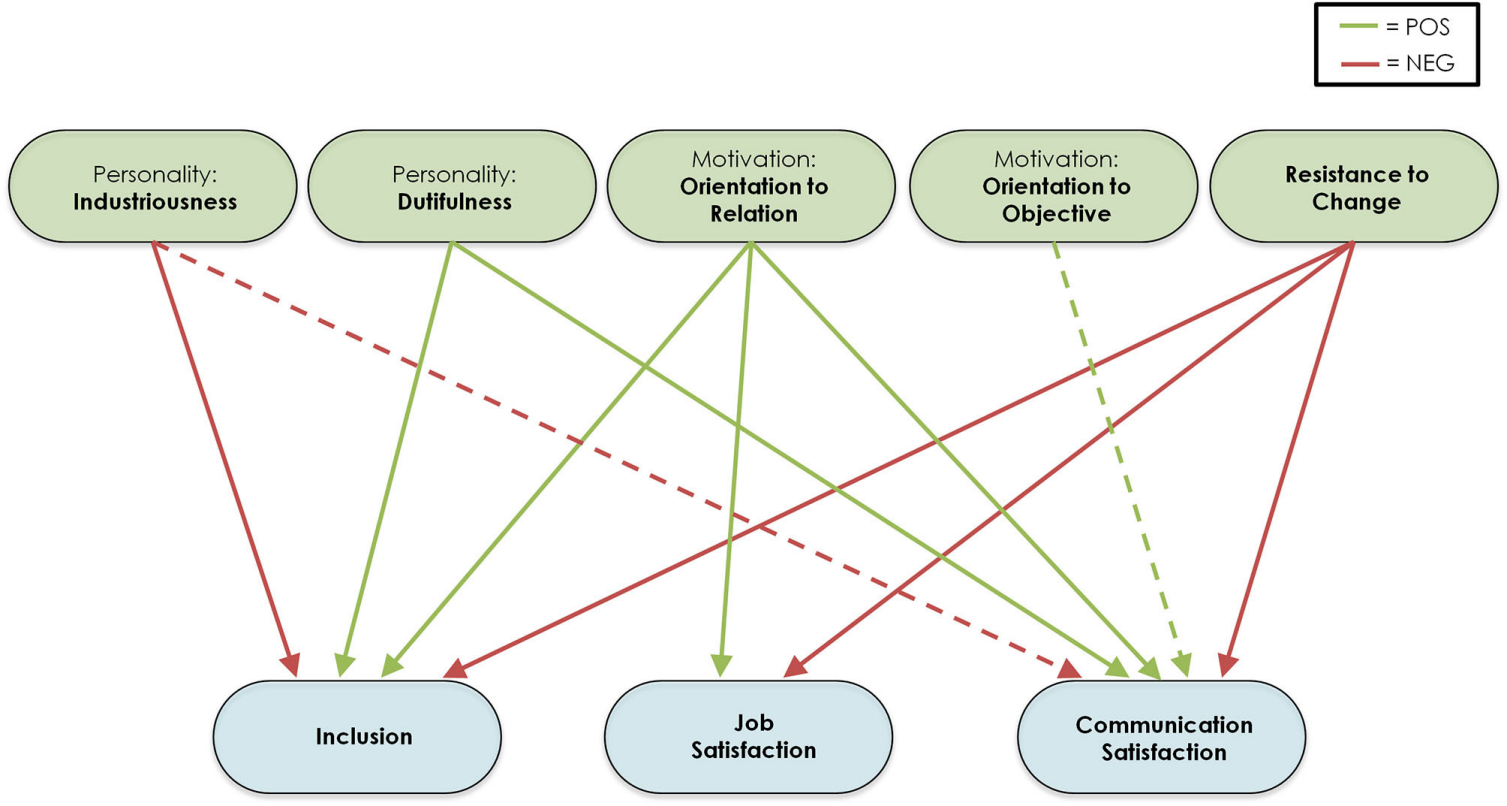
Summary of results from Structural Equation Models. This figure represents the statistically significant effects found across all Structural Equation Models tested. Green boxes represent exogenous (independent) variables measured in employees, while blue boxes represent endogenous (dependent) variables from employees. Green arrows represent statistically significant positive effects, red arrows represent statistically significant negative effects. Dashed lines represent two effects found as statistically significant in all tested models except for the model with reduced parameters (M4).

Statistically significant effects are reported in Table 2. Significant effects showed noteworthy consistency across different models (only two effects were not significant in M4) and are summarized in Figure 1. Orientation to relation showed a significant positive effect toward all three outcome measures, while resistance to change presented a significant negative effect toward all outcome variables. Therefore, employees higher in orientation to relation and lower in resistance to change showed better success in relocation. Industriousness showed significant negative effects toward inclusion and communication satisfaction, while dutifulness displayed significant positive effects toward the two same outcome variables. Finally, orientation to objective showed a significant positive effect toward communication satisfaction. This latter effect and the effect of industriousness toward communication satisfaction did not show to be statistically significant in model M4.

### Testing Sample

Predicted scores for the three outcome measures were computed in the testing sample (n = 79) according to the parameters found in models M3 and M4 (i.e., the two models with the lowest AIC, representing the largest explained variance with the lowest number of parameters). Predicted scores were then compared to observed scores to test the predictive accuracy of the models by using the Mean Absolute Error (MAE). MAE values for the two models M3 and M4 are reported in Table 3. Higher MAE values in M4 compared to M3 showed that prediction accuracy in the testing phase was higher in M3 than M4. This result indicates that the higher number of parameters in M3 contributed to predicting more accurately the outcome scores in the testing sample, thus generalizing better the results to an external dataset.

**Table 3:** Mean Absolute Error (MAE) in the testing sample in Structural Equation Models.

**MAE**	**M3**	**M4**
INQ_Inclusion	11.04	13.33
JSI_JobSatisfaction	10.54	16.97
CSS_CommunicationSatisfaction	10.00	13.46

## Machine Learning Algorithms

Like SEMs, supervisor-related feature variables have been dropped from Machine Learning models due to their multicollinearity. The rest of the features have all been included in the validation process of each model.

### Feature Selection

#### Inclusion

**Figure 2:**
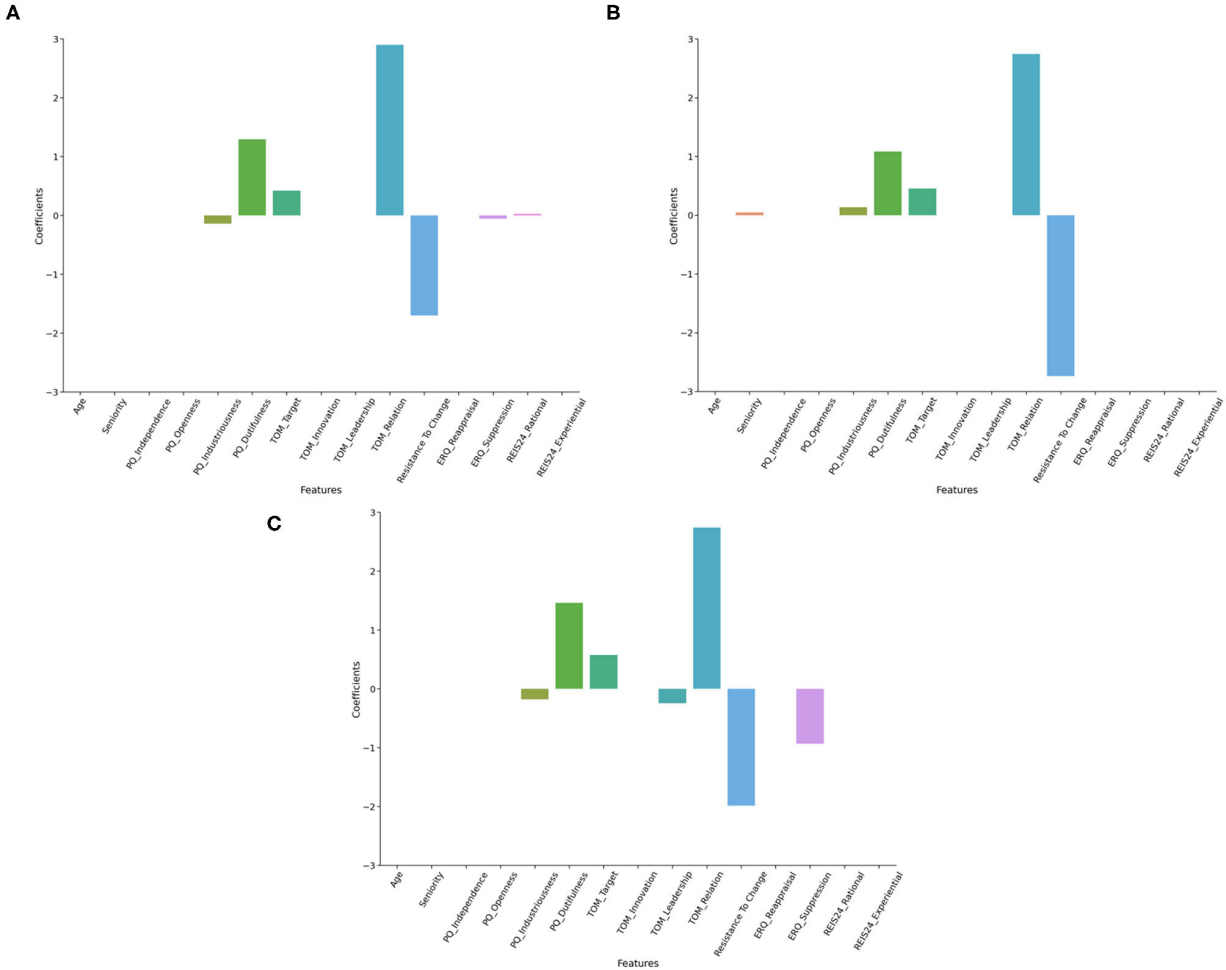
Feature coefficients in Lasso regressors. This figure represents the coefficients of features surviving the regularization in Lasso regressors. Models predicting inclusion **(A)**, job satisfaction **(B)**, and communication satisfaction **(C)** are represented.

For the inclusion target variable, the best Lasso Regression model (Alpha = 0.6, MAE = 9.71) reported a major influence of TOM Relation (2.9), Resistance to change (−1.7), PQ Dutifulness (1.3), TOM Target (0.42), PQ Industriousness (−0.14), ERQ Suppression (−0.06) and REI Rational (0.03—see Figure 2A).

#### Job Satisfaction

The best Lasso Regression model (Alpha = 0.6, MAE = 10.91) for the Job Satisfaction target variable showed a notable influence of TOM Relation (2.74), Resistance to change (−2.74), PQ Dutifulness (1.08), TOM Target (0.46), PQ Industriousness (0.14), and Seniority (0.05—see Figure 2B).

#### Communication Satisfaction

Considering as target variable the Communication Satisfaction, the best Lasso Regression model (Alpha = 0.6, MAE = 10.45) reported a major influence of TOM Relation (2.74), Resistance to change (−1.99), PQ Dutifulness (1.46), ERQ Suppression (−0.93), TOM Target (0.58), TOM Leadership (−0.24), PQ Industriousness (−0.18—see Figure 2C).

### Classification Models

#### Inclusion

The best K-Nearest Neighbor Classifier (weights = distance, n neighbors = 31 and metric = euclidean) reported a cross-validation accuracy score of the training set higher (0.66) compared to the test set (0.61). The prediction of the low inclusion class (1, N = 32) on the test set reached a precision of 0.52 and a recall of 0.50 (F1 score = 0.51). This was lower compared to the high inclusion class (2, N = 47) that scored 0.67 on precision and 0.68 on the recall (F1 score = 0.67).

**Figure 3:**
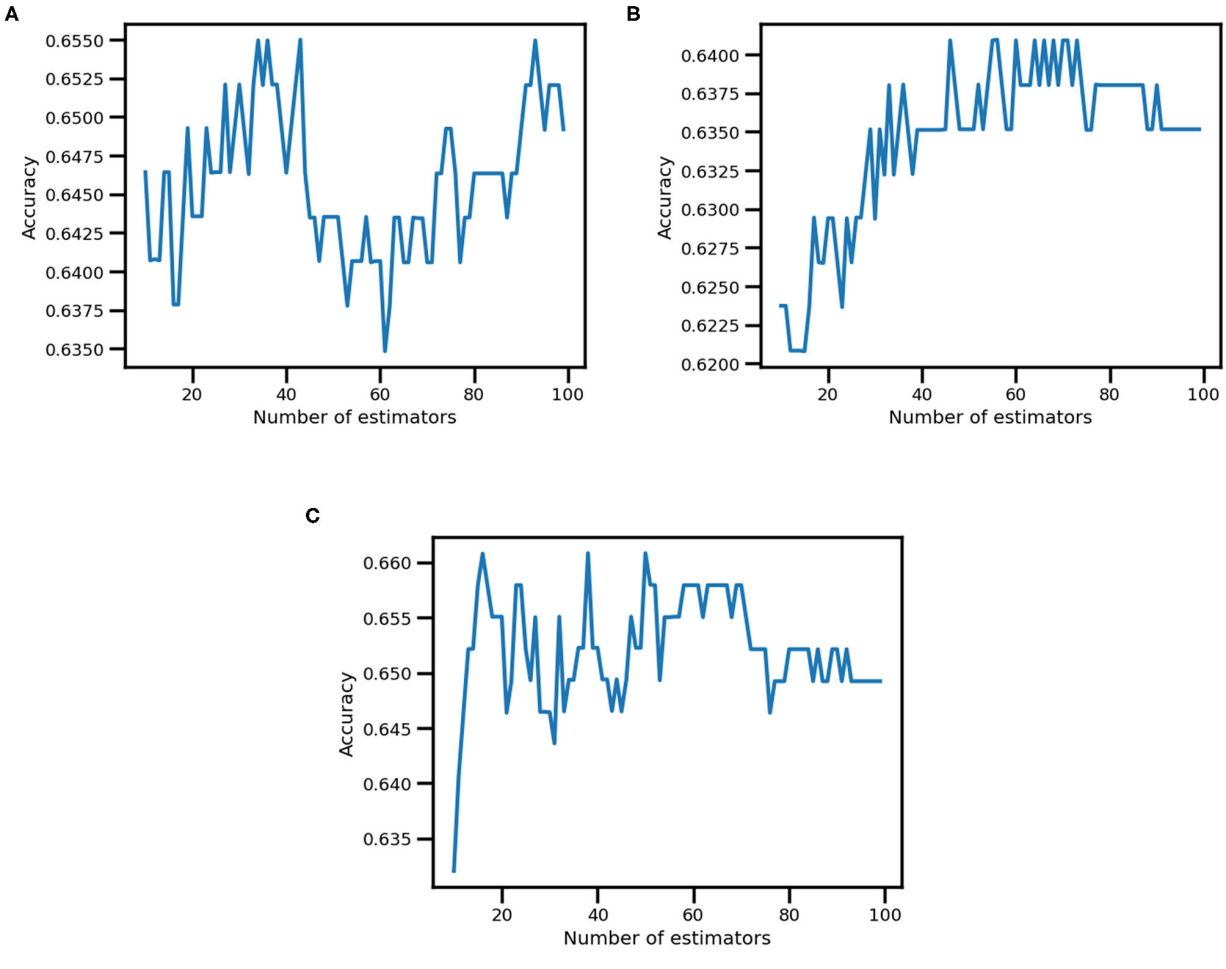
Accuracy in Ensemble learning *k*-NN. This figure represents the accuracy variation (on the y-axis) based on the number of estimators (i.e., number of classifiers included in the Bagging Classifier, on the x-axis) for inclusion **(A)**, job satisfaction **(B)**, and communication satisfaction **(C)** output variables.

The best Bagging Classifier (n estimators = 43—see Figure 3A) did not show overfitting or underfitting. Indeed, the cross-validation accuracy score of the training set was 0.65 ± 0.05 compared to 0.63 of the test set. Moreover, the Bagging Classifier slightly enhanced the prediction performance of both the low inclusion (precision = 0.55, recall = 0.53 and F1 score = 0.54) and the high inclusion class (precision = 0.60, recall = 0.70 and F1 score = 0.69) compared to the single K-Nearest Neighbor Classifier.

#### Job Satisfaction

The best K-Nearest Neighbor Classifier (weights = distance, n neighbors = 47 and metric = manhattan) reported a training set cross-validation accuracy score of 0.65 compared to 0.63 of the test set . Moreover, on the test set, the prediction performance of the low job satisfaction class (N = 28) scored lower (precision = 0.48, recall = 0.54 and F1 score = 0.51) compared to the high job satisfaction class (precision = 0.73, recall = 0.69 and F1 score = 0.71, N = 51).

The best Bagging Classifier (n estimators = 56—see Figure 3B) did not show overfitting or underfitting and displayed a slight increase in model performance. Accordingly, the cross-validation accuracy score of the training set was 0.64 ± 0.03, while of the test set was 0.66. Moreover, the Bagging Classifier slightly increased the prediction performance of both the low job satisfaction (precision = 0.52, recall = 0.54 and F1 score = 0.53) and the high job satisfaction class (precision = 0.74, recall = 0.73 and F1 score = 0.73) compared to the single K-Nearest Neighbor Classifier.

#### Communication Satisfaction

We observed that the best K-Nearest Neighbor Classifier (weights = uniform, n neighbors = 59 and metric = euclidean) reported a higher cross-validation accuracy score of the training set (0.66) compared to the test set (0.65). Again, on the test set, the prediction performance of the low communication satisfaction class (N = 32) scored lower (precision = 0.56, recall = 0.56 and F1 score = 0.56) compared to the high communication satisfaction class (precision = 0.70, recall = 0.70 and F1 score = 0.70, N = 47).

The best Bagging Classifier (n estimators = 38—see Figure 3C) did not show overfitting or underfitting, but a small increment in model performance. Indeed, the cross-validation accuracy score of the training set reached 0.66 ± 0.04, while of the test set was 0.66. Moreover, the Bagging Classifier increased the prediction performance of both the low communication satisfaction class (precision = 0.58, recall = 0.56 and F1 score = 0.57) and the high communication satisfaction class (precision = 0.71, recall = 0.72 and F1 score = 0.72) compared to the single K-Nearest Neighbor Classifier.

## Discussion

Internal mobility has been previously investigated as a specific form of job relocation. Nevertheless, no studies have identified what characteristics (in both employees and supervisors) can predict successful mobility in terms of job satisfaction. In this study, we compared different classes of models to identify the most efficient technique to predict successful mobility, i.e., (i) traditional Structural Equation Modeling (SEM), with two families of Machine Learning algorithms: (ii) regressors, specifically least absolute shrinkage and selection operator (Lasso) aimed at feature selection and (iii) classifiers, specifically *k*-nearest neighbors algorithm (*k*-NN). Results showed different performances for the three classes of models, ranging from low to medium accuracy.

A crucial aspect in results is the consistency among statistically significant effects. All SEMs replicated the statistical significance of the effects involving five predictors: (i) orientation to relation and resistance to change showed to influence all endogenous variables with high effect sizes, (ii) dutifulness, industriousness and orientation to objective displayed a relevant but less consistent contribution, as they did not influence all endogenous variables, and in the reduced model (M4) two of the parameters appeared not to be statistically significant. A decisive result is a consistency with the Lasso results, in particular for what concerns orientation to relation and resistance to change. Minor results also identified employees' seniority, rational style, expressive suppression and orientation to leadership as relevant in Lasso models. Feature selection in Lasso models allows predicting the target variable by zeroing the coefficient of the less important features and using only the remaining ones in the model. The fact that two models stemming from different approaches to data analysis replicated comparable results is acceptable evidence for results consistency.

Previous literature showed that personality dispositions, resistance to change and social orientations are crucial for mobility relocation (Otto and Dalbert, 2012), and managing resistance to change in the workplace appears to be fundamental to stimulate job satisfaction (Laframboise et al., 2003). Resistance to change is defined as an individual's dispositional inclination to resist changes (declined in routine seeking, emotional reaction to imposed change, cognitive rigidity, and short-term focus, Oreg, 2003) and therefore the influence of this construct on job relocation appears to be self-evident. Nevertheless, having measured the influence of this variable on all three indices of successful relocation with such consistency is meaningful evidence for the robustness of this relation. At the same time, the orientation to social relation was previously shown to be fundamental in some careers with a great number of employee-customer exchanges (Alessandri and Russo, 2011). According to our data, orientation to relation appears to be crucial in successful relocation for any employee, and not only for a specific personality phenotype as previously found (Otto and Dalbert, 2012). This result shows that motivational orientation toward social bonding is crucial when a change in the social group (i.e., job relocation) is experienced. This motivational orientation can stimulate a person to be accepted by the new social group and, consequently, experience higher job satisfaction in the workplace.

In some models we reported, industriousness predicted negatively successful relocation. This result is related to the fact that the industriousness facet captured several aspects related to workaholism (e.g., being under constant pressure, putting work before pleasure) (Jackson et al., 1996a), that can be considered as the extreme opposite of job satisfaction (Burke, 2001). Dutifulness appears to be relevant for a successful relocation, as it explains aspects related to prudence and compliance with rules, which are fundamental for inclusion in a new social group. Finally, the suppression emotion regulation strategy negatively predicts success in job relocation in some Lasso models, as it represents a basic regulation strategy, which is often not effective in intrapersonal and interpersonal functioning (Gross and John, 2003).

In SEMs, M3 and M4 are the most efficient models, considering only exogenous variables from employees. When choosing the best model, on the one hand, the likelihood ratio test (LRT) in the training set did not find a significant difference in explained variance. This result favored M4, as it explained a comparable level of variance with an extremely lower number of parameters (as displayed by lower AIC). On the other hand, validation on the test set appeared to favor M3, as it showed lower MAE values than M4 across all three endogenous variables. Therefore, the higher number of parameters in M3 occurred to describe better data in the test set. To sum up, we cannot univocally prefer one of the two models, as M3 generalized better results on the test set, while M4 performed more efficiently on the training set. On the contrary, models M1 and M2 (the least efficient ones, in terms of AIC) showed that supervisors' data, as well as age and seniority, are not relevant in predicting success in relocation. The interpretation of these (null) results would imply low relevance of the supervisors' role in relocation success; nevertheless, we cannot interpret this result because multicollinearity generated by using aggregate data for supervisors could invalidate them. Moreover, there is a substantial overlap between the features surviving in Lasso regressors and statistically significant exogenous variables in SEMs. Since in Lasso models only the most relevant features survive, the meaningful agreement between these two classes of models shows the statistical relevance of these effects and the reliability of the results.

When chunking the information with feature selection and classifying high vs. low relocation success (i.e., job satisfaction, inclusion and communication satisfaction) by means of *k*-NN algorithms, it was possible to predict success with 64–66% accuracy in the training set and 61–65% in the test set, which represent medium performance. Overall, bagging meta-models displayed slightly higher performances compared to the single *k*-NNs and, despite the small dataset, they did not show underfitting or overfitting. The ensemble meta-model reached a 64–66% accuracy in the training set and a 63–66% on the test set, raising also the F1 score of each predicted class. Indeed, bagging is based on bootstrapping techniques, which is frequently used with small datasets. This represents further evidence that results could be improved by using larger datasets in both training and testing phases (see next paragraph). These results also show that the algorithms often generalized well on the test set. However, the algorithms showed to predict high success in relocation (values above the median) with generally higher precision and recall than low success (below the median). An explanation of this result can be related to the relatively small sample size, which was generally biased toward high scores (i.e., more participants with high values in the three outcomes than with low values). Because of this imbalance in classes, algorithms were most probably better trained on identifying participants with high relocation success than low success.

In summary, our results suggest that SEMs (more broadly used in HR literature, Borgogni et al., 2010) can estimate successful relocation with average accuracy. Resistance to change and orientation to relation were found to be the most relevant predictors, as confirmed by Lasso regressors. Bagging Classifier with *k*-NN as base estimator displayed good performance in classifying data, showing potentiality in using machine learning techniques in predictive HR analytics. The performance of these algorithms could be increased in future research by increasing sample size and including further predictors, as specified hereafter.

### Limitations

The main methodological limitation of this study is represented by having used aggregate data for supervisors. Unfortunately, for privacy reasons, this was our better option, since we could trace supervisors back to Business Units, but not to individual direct reports. These aggregate data generated multicollinearity in both SEMs and machine learning algorithms using data from supervisors, thus making null effects in these predictors hard to interpret. The lack of influence from supervisors' features on the relocation success in employees would be an outstanding result in terms of implications, but, unfortunately, this interpretation is impractical for methodological limitations.

Despite a discretely large sample size in both training and testing sets, this study could have benefitted from larger samples. The main reason is the large number of predictors involved in models, which would need an adequate number of participants in order to fit the data (Sawyer, 1982; Fursov et al., 2018).

Another theoretical limitation of our study is the fact that we considered only post-relocation data. Dependent and independent variables have been measured only after the transfer had occurred (more than 6 months before data collection). Hence, we do not have information about such variables before the transfer, that is, we do not know the level of job satisfaction, inclusion, and communication satisfaction related to the old job position. Hence, we cannot exclude that there are differences in job satisfaction between old and new positions inherently related to the specificities of the new job position. Actually, in our study, we adopted the point of view of such practitioners, interested in estimating future relocation success for normative purposes.

Different classification methods could have been chosen instead of K-Nearest Neighbors. In our specific case, these alternative methods would have yielded similar results (Supplementary Material). This aspect may represent a future development of this study, consisting in comparing different algorithms in several fields of predictive HR analytics according to different research or market questions.

Future directions for this study may also consider adding predictors which are known to be predictive of satisfaction in relocation. These predictors would include both stable psychological traits (e.g., personality factors) and social-environmental features (e.g., economic conditions, family characteristics, differences among industries and occupations).

### Conclusions

To sum up, we found that traditional SEMs predicted with average accuracy successful relocation, thus identifying the relevance of resistance to change and orientation to relation. Lasso regressors confirmed the influence of these variables; while *k*-NN classifiers displayed good performance in classifying data.

The practical application of these results is prominent in the field of HR, as we have empirical evidence for pushing for training employees who are going to be relocated in reducing their resistance to change, thus promoting resilience, and improving their social skills, aside from training in hard skills. Moreover, we show that artificial intelligence algorithms could help in selecting employees who are more prone to be relocated to a new job position, with all due ethical reservations and in conjunction with further methods such as interviews, validated questionnaires, et cetera.

In the field of predictive HR analytics, this is a seminal result comparing methods stemming from different research traditions. There is considerable room for improvement since the models' efficiency was typically not high. Future research will have to consider different variables and different approaches, but we believe it is crucial to start comparing the performance from divergent methods. The aim of this comparison is not to find that one method is better than others in the entire field of HR analytics, but to make them all available and comparable according to different research (and market) questions.

## Data Availability Statement

The raw data supporting the conclusions of this article will be made available by the authors, without undue reservation.

## Ethics Statement

The studies involving human participants were reviewed and approved by Area Vasta Nord Ovest Ethics Committee. The patients/participants provided their written informed consent to participate in this study.

## Author Contributions

FB, AMal, and ER contributed to the design, the conception of the research, and contributed to the manuscript revision. FB contributed to the implementation of the study and data collection. FB and FD contributed to analyzing data. FB, FD, and AMas contributed to writing the manuscript. SD'A as a member of Intesa Sanpaolo Innovation Center S.p.A., assisted with the project management between IMT School for Advanced Studies Lucca and Intesa Sanpaolo Group. All authors contributed to the article, read, and approved the submitted version.

## Funding

The authors declare that this study received funding from Intesa Sanpaolo Innovation Center S.p.A. The funder was not involved in the study design, collection, analysis, interpretation of data, and the writing of this article or the decision to submit it for publication.

## Conflict of Interest

SD'A was employed by Intesa Sanpaolo Innovation Center S.p.A. The remaining authors declare that the research was conducted in the absence of any commercial or financial relationships that could be construed as a potential conflict of interest.

## Publisher's Note

All claims expressed in this article are solely those of the authors and do not necessarily represent those of their affiliated organizations, or those of the publisher, the editors and the reviewers. Any product that may be evaluated in this article, or claim that may be made by its manufacturer, is not guaranteed or endorsed by the publisher.
