# Supplementary material for: Estimating Successful Internal Mobility: A Comparison Between Structural Equation Models and Machine Learning Algorithms
Source: Front Artif Intell. 2022 Mar 25;5:848015. doi: 10.3389/frai.2022.848015 (PMC8990773; doi:10.3389/frai.2022.848015)
Supplement: Supplementary file 1 [file Table_1.DOCX]

# SUPPLEMENTARY MATERIALS

*Acronyms for all tables:* INQ = Inclusion Questionnaire; JSI = Job Satisfaction Index; CSS = Communication Satisfaction Scale; PQ = Personality Questionnaire; TOM = Motivational Orientation Test (original name: Test di Orientamento Motivazionale); ERQ = Emotion Regulation Questionnaire; REIS24 = Rational-Experiential Inventory – Short form 24 items; TEIQ = Trait Emotional Intelligence Questionnaire; IRI = Interpersonal Reactivity Instrument; PSA = Prosocialness Scale for Adults; *k*-NN = K-Nearest Neighbors; SVC = Support Vector Classifier (polynomial kernel); DT = Decision Tree

## Structural Equation Models: Parameters

*Acronym:* Std. lv = effects estimate standardized on the first manifest variable; in our models, this corresponds to the default estimate. Std. all = effects estimate standardized on all manifest variables.

| M1 | | | | | | |
| --- | --- | --- | --- | --- | --- | --- |
| **REGRESSIONS** | | | | | | |
|  | **Estimate** | **Std. Err** | **z value** | **P (>\|z\|)** | **Std. lv** | **Std. all** |
| INQ_Inclusion ~ | | | | | | |
| PQ_Independence | -0.159 | 0.241 | -0.661 | 0.509 | -0.159 | -0.040 |
| PQ_Openness | 0.144 | 0.198 | 0.726 | 0.468 | 0.144 | 0.054 |
| **PQ_Industriousness** | **-0.422** | **0.182** | **-2.316** | **0.021** | **-0.422** | **-0.137** |
| **PQ_Dutifulness** | **0.439** | **0.173** | **2.534** | **0.011** | **0.439** | **0.149** |
| TOM_Target | 0.261 | 0.164 | 1.596 | 0.111 | 0.261 | 0.150 |
| TOM_Innovation | -0.215 | 0.160 | -1.347 | 0.178 | -0.215 | -0.136 |
| TOM_Leadership | 0.004 | 0.086 | 0.052 | 0.958 | 0.004 | 0.003 |
| **TOM_Relation** | **0.577** | **0.176** | **3.271** | **0.001** | **0.577** | **0.220** |
| **Resistance to Change** | **-0.170** | **0.077** | **-2.192** | **0.028** | **-0.170** | **-0.147** |
| ERQ_Reappraisal | 0.051 | 0.162 | 0.315 | 0.753 | 0.051 | 0.017 |
| ERQ_Suppression | -0.213 | 0.188 | -1.129 | 0.259 | -0.213 | -0.059 |
| REIS24_Rational | 0.104 | 0.115 | 0.900 | 0.368 | 0.104 | 0.054 |
| REIS24_Experiential | 0.043 | 0.099 | 0.435 | 0.664 | 0.043 | 0.023 |
| *Supervisors*:  TEIQ_Emotional Intelligence | 0.059 | 0.958 | 0.061 | 0.951 | 0.059 | 0.025 |
| *Supervisors*:  IRI_Empathy | 0.295 | 1.241 | 0.238 | 0.812 | 0.295 | 0.072 |
| *Supervisors*:  PSA_Prosociality | -1.169 | 2.799 | -0.417 | 0.676 | -1.169 | -0.140 |
| *Supervisors*:  REIS24_Rational | 0.105 | 1.624 | 0.064 | 0.949 | 0.105 | 0.016 |
| *Supervisors*:  REIS24_Experiential | -1.555 | 3.369 | -0.461 | 0.644 | -1.555 | -0.127 |
|  | | | | | | |
|  | **Estimate** | **Std. Err** | **z value** | **P (>\|z\|)** | **Std. lv** | **Std. all** |
| JSI_JobSatisfaction ~ | | | | | | |
| PQ_Independence | -0.019 | 0.261 | -0.072 | 0.943 | -0.019 | -0.004 |
| PQ_Openness | 0.275 | 0.214 | 1.280 | 0.200 | 0.275 | 0.095 |
| PQ_Industriousness | -0.030 | 0.197 | -0.151 | 0.880 | -0.030 | -0.009 |
| PQ_Dutifulness | 0.303 | 0.188 | 1.615 | 0.106 | 0.303 | 0.095 |
| TOM_Target | 0.281 | 0.177 | 1.586 | 0.113 | 0.281 | 0.148 |
| TOM_Innovation | -0.284 | 0.173 | -1.641 | 0.101 | -0.284 | -0.165 |
| TOM_Leadership | 0.023 | 0.093 | 0.251 | 0.802 | 0.023 | 0.015 |
| **TOM_Relation** | **0.560** | **0.191** | **2.929** | **0.003** | **0.560** | **0.196** |
| **Resistance to Change** | **-0.287** | **0.084** | **-3.420** | **0.001** | **-0.287** | **-0.227** |
| ERQ_Reappraisal | 0.040 | 0.176 | 0.229 | 0.819 | 0.040 | 0.013 |
| ERQ_Suppression | -0.115 | 0.204 | -0.564 | 0.573 | -0.115 | -0.029 |
| REIS24_Rational | -0.081 | 0.125 | -0.652 | 0.514 | -0.081 | -0.039 |
| REIS24_Experiential | -0.092 | 0.107 | -0.861 | 0.389 | -0.092 | -0.044 |
| *Supervisors*:  TEIQ_Emotional Intelligence | -1.056 | 1.038 | -1.017 | 0.309 | -1.056 | -0.413 |
| *Supervisors*:  IRI_Empathy | -1.896 | 1.345 | -1.410 | 0.159 | -1.896 | -0.424 |
| *Supervisors*:  PSA_Prosociality | 3.846 | 3.033 | 1.268 | 0.205 | 3.846 | 0.422 |
| *Supervisors*:  REIS24_Rational | 0.685 | 1.759 | 0.389 | 0.697 | 0.685 | 0.098 |
| *Supervisors*:  REIS24_Experiential | 2.186 | 3.650 | 0.599 | 0.549 | 2.186 | 0.164 |
|  | | | | | | |
|  | **Estimate** | **Std. Err** | **z value** | **P (>\|z\|)** | **Std. lv** | **Std. all** |
| CSS_CommunicationSatisfaction~ | | | | | | |
| PQ_Independence | 0.075 | 0.256 | 0.294 | 0.769 | 0.075 | 0.018 |
| PQ_Openness | 0.054 | 0.211 | 0.255 | 0.799 | 0.054 | 0.019 |
| **PQ_Industriousness** | **-0.410** | **0.194** | **-2.115** | **0.034** | **-0.410** | **-0.125** |
| **PQ_Dutifulness** | **0.429** | **0.184** | **2.327** | **0.020** | **0.429** | **0.137** |
| **TOM_Target** | **0.384** | **0.174** | **2.207** | **0.027** | **0.384** | **0.207** |
| TOM_Innovation | -0.192 | 0.170 | -1.128 | 0.259 | -0.192 | -0.114 |
| TOM_Leadership | -0.123 | 0.091 | -1.346 | 0.178 | -0.123 | -0.080 |
| **TOM_Relation** | **0.647** | **0.188** | **3.446** | **0.001** | **0.647** | **0.231** |
| **Resistance to Change** | **-0.214** | **0.082** | **-2.597** | **0.009** | **-0.214** | **-0.173** |
| ERQ_Reappraisal | -0.138 | 0.173 | -0.799 | 0.424 | -0.138 | -0.044 |
| ERQ_Suppression | -0.369 | 0.200 | -1.839 | 0.066 | -0.369 | -0.369 |
| REIS24_Rational | 0.009 | 0.123 | 0.071 | 0.943 | 0.009 | 0.004 |
| REIS24_Experiential | 0.034 | 0.105 | 0.326 | 0.744 | 0.034 | 0.017 |
| *Supervisors*:  TEIQ_Emotional Intelligence | -1.248 | 1.020 | -1.224 | 0.221 | -1.248 | 0.500 |
| *Supervisors*:  IRI_Empathy | 0.173 | 1.321 | 0.131 | 0.896 | 0.173 | 0.040 |
| *Supervisors*:  PSA_Prosociality | -0.258 | 2.979 | -0.086 | 0.931 | -0.258 | -0.029 |
| *Supervisors*:  REIS24_Rational | 1.895 | 1.728 | 1.096 | 0.273 | 1.895 | 0.277 |
| *Supervisors*:  REIS24_Experiential | 4.142 | 3.585 | 1.155 | 0.248 | 4.142 | 0.318 |
|  | | | | | | |
| **COVARIANCES** | | | | | | |
|  | **Estimate** | **Std. Err** | **z value** | **P (>\|z\|)** | **Std. lv** | **Std. all** |
| INQ_Inclusion ~~ | | | | | | |
| **JSI_JobSatisfaction** | **77.390** | **9.336** | **8.290** | **< 0.001** | **77.390** | **0.496** |
| **CSS_CommunicationSatisfaction** | **84.834** | **9.390** | **9.035** | **< 0.001** | **84.834** | **0.554** |
| JSI_JobSatisfaction ~~ | | | | | | |
| **CSS_CommunicationSatisfaction** | **74.920** | **9.764** | **7.673** | **< 0.001** | **74.920** | **0.451** |
|  | | | | | | |
| **VARIANCES** | | | | | | |
|  | **Estimate** | **Std. Err** | **z value** | **P (>\|z\|)** | **Std. lv** | **Std. all** |
| **INQ_Inclusion** | **144.008** | **10.917** | **13.191** | **< 0.001** | **144.008** | **0.827** |
| **JSI_JobSatisfaction** | **169.020** | **12.813** | **13.191** | **< 0.001** | **169.020** | **0.815** |
| **CSS_CommunicationSatisfaction** | **163.083** | **12.363** | **13.191** | **< 0.001** | **163.083** | **0.824** |

| M2 | | | | | | |
| --- | --- | --- | --- | --- | --- | --- |
| **REGRESSIONS** | | | | | | |
|  | **Estimate** | **Std. Err** | **z value** | **P (>\|z\|)** | **Std. lv** | **Std. all** |
| INQ_Inclusion ~ | | | | | | |
| PQ_Independence | -0.082 | 0.243 | -0.338 | 0.736 | -0.082 | -0.021 |
| PQ_Openness | 0.130 | 0.200 | 0.650 | 0.516 | 0.130 | 0.049 |
| **PQ_Industriousness** | **-0.418** | **0.182** | **-2.300** | **0.021** | **-0.418** | **-0.136** |
| **PQ_Dutifulness** | **0.444** | **0.174** | **2.555** | **0.011** | **0.444** | **0.151** |
| TOM_Target | 0.247 | 0.164 | 1.502 | 0.133 | 0.247 | 0.142 |
| TOM_Innovation | -0.222 | 0.161 | -1.381 | 0.167 | -0.222 | -0.140 |
| TOM_Leadership | -0.002 | 0.086 | -0.019 | 0.984 | -0.002 | -0.001 |
| **TOM_Relation** | **0.615** | **0.179** | **3.438** | **0.001** | **0.615** | **0.234** |
| **Resistance to Change** | **-0.188** | **0.077** | **-2.448** | **0.014** | **-0.188** | **-0.162** |
| ERQ_Reappraisal | 0.028 | 0.163 | 0.169 | 0.865 | 0.028 | 0.009 |
| ERQ_Suppression | -0.182 | 0.190 | -0.960 | 0.337 | -0.182 | -0.051 |
| REIS24_Rational | 0.088 | 0.115 | 0.765 | 0.444 | 0.088 | 0.046 |
| REIS24_Experiential | 0.053 | 0.099 | 0.529 | 0.597 | 0.053 | 0.028 |
| Age | -0.266 | 0.145 | -1.837 | 0.066 | -0.266 | -0.140 |
| Seniority | 0.224 | 0.121 | 1.848 | 0.065 | 0.224 | 0.139 |
| *Supervisors*: Age | -0.081 | 0.302 | -0.269 | 0.788 | -0.081 | -0.028 |
| *Supervisors*: Seniority | 0.146 | 0.274 | 0.534 | 0.593 | 0.146 | 0.056 |
|  | | | | | | |
|  | **Estimate** | **Std. Err** | **z value** | **P (>\|z\|)** | **Std. lv** | **Std. all** |
| JSI_JobSatisfaction ~ | | | | | | |
| PQ_Independence | -0.001 | 0.266 | -0.003 | 0.998 | -0.001 | -0.000 |
| PQ_Openness | 0.238 | 0.219 | 1.087 | 0.277 | 0.238 | 0.082 |
| PQ_Industriousness | 0.058 | 0.199 | 0.292 | 0.770 | 0.058 | 0.017 |
| PQ_Dutifulness | 0.282 | 0.190 | 1.480 | 0.139 | 0.282 | 0.088 |
| TOM_Target | 0.266 | 0.180 | 1.477 | 0.140 | 0.266 | 0.140 |
| TOM_Innovation | -0.292 | 0.176 | -1.656 | 0.098 | -0.292 | -0.169 |
| TOM_Leadership | 0.026 | 0.094 | 0.273 | 0.785 | 0.026 | 0.016 |
| **TOM_Relation** | **0.593** | **0.196** | **3.028** | **0.002** | **0.593** | **0.207** |
| **Resistance to Change** | **-0.323** | **0.084** | **-3.846** | **< 0.001** | **-0.323** | **-0.255** |
| ERQ_Reappraisal | 0.014 | 0.179 | 0.080 | 0.936 | 0.014 | 0.004 |
| ERQ_Suppression | -0.093 | 0.208 | -0.450 | 0.653 | -0.093 | -0.024 |
| REIS24_Rational | -0.098 | 0.127 | -0.773 | 0.440 | -0.098 | -0.047 |
| REIS24_Experiential | -0.081 | 0.109 | -0.746 | 0.455 | -0.081 | -0.039 |
| Age | -0.138 | 0.159 | -0.866 | 0.386 | -0.138 | -0.066 |
| Seniority | 0.189 | 0.133 | 1.424 | 0.154 | 0.189 | 0.107 |
| *Supervisors*: Age | -0.106 | 0.331 | -0.321 | 0.748 | -0.106 | -0.034 |
| *Supervisors*: Seniority | 0.199 | 0.300 | 0.662 | 0.508 | 0.199 | 0.069 |
|  | | | | | | |
|  | **Estimate** | **Std. Err** | **z value** | **P (>\|z\|)** | **Std. lv** | **Std. all** |
| CSS_CommunicationSatisfaction~ | | | | | | |
| PQ_Independence | 0.097 | 0.258 | 0.375 | 0.708 | 0.097 | 0.023 |
| PQ_Openness | 0.060 | 0.212 | 0.283 | 0.777 | 0.060 | 0.021 |
| **PQ_Industriousness** | **-0.413** | **0.193** | **-2.137** | **0.033** | **-0.413** | **-0.126** |
| **PQ_Dutifulness** | **0.439** | **0.185** | **2.378** | **0.017** | **0.439** | **0.140** |
| **TOM_Target** | **0.379** | **0.175** | **2.169** | **0.030** | **0.379** | **0.204** |
| TOM_Innovation | -0.180 | 0.171 | -1.055 | 0.291 | -0.180 | -0.107 |
| TOM_Leadership | -0.129 | 0.091 | -1.415 | 0.157 | -0.129 | -0.084 |
| **TOM_Relation** | **0.664** | **0.190** | **3.493** | **< 0.001** | **0.664** | **0.237** |
| **Resistance to Change** | **-0.212** | **0.081** | **-2.605** | **0.009** | **-0.212** | **-0.172** |
| ERQ_Reappraisal | -0.157 | 0.173 | -0.903 | 0.367 | -0.157 | -0.050 |
| ERQ_Suppression | -0.371 | 0.201 | -1.841 | 0.066 | -0.371 | -0.097 |
| REIS24_Rational | -0.001 | 0.123 | -0.011 | 0.991 | -0.001 | -0.001 |
| REIS24_Experiential | 0.037 | 0.105 | 0.350 | 0.727 | 0.037 | 0.018 |
| Age | -0.134 | 0.154 | -0.873 | 0.383 | -0.134 | -0.066 |
| Seniority | 0.112 | 0.129 | 0.870 | 0.384 | 0.112 | 0.065 |
| *Supervisors*: Age | -0.322 | 0.321 | -1.004 | 0.315 | -0.322 | -0.104 |
| *Supervisors*: Seniority | 0.351 | 0.291 | 1.206 | 0.228 | 0.351 | 0.125 |
|  | | | | | | |
| **COVARIANCES** | | | | | | |
|  | **Estimate** | **Std. Err** | **z value** | **P (>\|z\|)** | **Std. lv** | **Std. all** |
| INQ_Inclusion ~~ | | | | | | |
| **JSI_JobSatisfaction** | **79.249** | **9.502** | **8.340** | **< 0.001** | **79.249** | **0.500** |
| **CSS_CommunicationSatisfaction** | **84.472** | **9.402** | **8.984** | **< 0.001** | **84.472** | **0.550** |
| JSI_JobSatisfaction ~~ | | | | | | |
| **CSS_CommunicationSatisfaction** | **75.594** | **9.897** | **7.638** | **< 0.001** | **75.594** | **0.449** |
|  | | | | | | |
| **VARIANCES** | | | | | | |
|  | **Estimate** | **Std. Err** | **z value** | **P (>\|z\|)** | **Std. lv** | **Std. all** |
| **INQ_Inclusion** | **144.682** | **10.968** | **13.191** | **< 0.001** | **144.682** | **0.831** |
| **JSI_JobSatisfaction** | **173.757** | **13.173** | **13.191** | **< 0.001** | **173.757** | **0.838** |
| **CSS_CommunicationSatisfaction** | **163.302** | **12.380** | **13.191** | **< 0.001** | **163.302** | **0.825** |

| M3 | | | | | | |
| --- | --- | --- | --- | --- | --- | --- |
| **REGRESSIONS** | | | | | | |
|  | **Estimate** | **Std. Err** | **z value** | **P (>\|z\|)** | **Std. lv** | **Std. all** |
| INQ_Inclusion ~ | | | | | | |
| PQ_Independence | -0.137 | 0.242 | -0.567 | 0.570 | -0.137 | -0.034 |
| PQ_Openness | 0.090 | 0.197 | 0.458 | 0.647 | 0.090 | 0.034 |
| **PQ_Industriousness** | **-0.368** | **0.180** | **-2.044** | **0.041** | **-0.368** | **-0.119** |
| **PQ_Dutifulness** | **0.421** | **0.174** | **2.422** | **0.015** | **0.421** | **0.143** |
| TOM_Target | 0.242 | 0.164 | 1.474 | 0.140 | 0.242 | 0.139 |
| TOM_Innovation | -0.217 | 0.161 | -1.350 | 0.177 | -0.217 | -0.137 |
| TOM_Leadership | 0.004 | 0.086 | 0.049 | 0.961 | 0.004 | 0.003 |
| **TOM_Relation** | **0.598** | **0.177** | **3.369** | **0.001** | **0.598** | **0.228** |
| **Resistance to Change** | **-0.187** | **0.077** | **-2.433** | **0.015** | **-0.187** | **-0.161** |
| ERQ_Reappraisal | 0.052 | 0.164 | 0.320 | 0.749 | 0.052 | 0.018 |
| ERQ_Suppression | -0.188 | 0.189 | -0.995 | 0.320 | -0.188 | -0.052 |
| REIS24_Rational | 0.096 | 0.116 | 0.826 | 0.409 | 0.096 | 0.050 |
| REIS24_Experiential | 0.035 | 0.099 | 0.349 | 0.727 | 0.035 | 0.018 |
|  | | | | | | |
|  | **Estimate** | **Std. Err** | **z value** | **P (>\|z\|)** | **Std. lv** | **Std. all** |
| JSI_JobSatisfaction ~ | | | | | | |
| PQ_Independence | -0.021 | 0.264 | -0.080 | 0.936 | -0.021 | -0.005 |
| PQ_Openness | 0.177 | 0.216 | 0.821 | 0.411 | 0.177 | 0.061 |
| PQ_Industriousness | 0.088 | 0.197 | 0.449 | 0.654 | 0.088 | 0.026 |
| PQ_Dutifulness | 0.266 | 0.190 | 1.398 | 0.162 | 0.266 | 0.083 |
| TOM_Target | 0.255 | 0.180 | 1.419 | 0.156 | 0.255 | 0.134 |
| TOM_Innovation | -0.273 | 0.176 | -1.555 | 0.120 | -0.273 | -0.158 |
| TOM_Leadership | 0.027 | 0.094 | 0.283 | 0.777 | 0.027 | 0.017 |
| **TOM_Relation** | **0.606** | **0.194** | **3.120** | **0.002** | **0.606** | **0.211** |
| **Resistance to Change** | **-0.320** | **0.084** | **-3.804** | **< .001** | **-0.320** | **-0.253** |
| ERQ_Reappraisal | 0.025 | 0.179 | 0.139 | 0.890 | 0.025 | 0.008 |
| ERQ_Suppression | -0.074 | 0.207 | -0.358 | 0.721 | -0.074 | -0.019 |
| REIS24_Rational | -0.096 | 0.127 | -0.757 | 0.449 | -0.096 | -0.046 |
| REIS24_Experiential | -0.102 | 0.108 | -0.936 | 0.349 | -0.102 | -0.049 |
|  | | | | | | |
|  | **Estimate** | **Std. Err** | **z value** | **P (>\|z\|)** | **Std. lv** | **Std. all** |
| CSS_CommunicationSatisfaction~ | | | | | | |
| PQ_Independence | 0.084 | 0.256 | 0.327 | 0.744 | 0.084 | 0.020 |
| PQ_Openness | 0.036 | 0.209 | 0.171 | 0.864 | 0.036 | 0.013 |
| **PQ_Industriousness** | **-0.401** | **0.191** | **-2.099** | **0.036** | **-0.401** | **-0.122** |
| **PQ_Dutifulness** | **0.429** | **0.184** | **2.330** | **0.020** | **0.429** | **0.137** |
| **TOM_Target** | **0.384** | **0.174** | **2.202** | **0.028** | **0.384** | **0.206** |
| TOM_Innovation | -0.173 | 0.170 | -1.018 | 0.309 | -0.173 | -0.103 |
| TOM_Leadership | -0.127 | 0.091 | -1.397 | 0.162 | -0.127 | -0.083 |
| **TOM_Relation** | **0.652** | **0.188** | **3.466** | **0.001** | **0.652** | **0.233** |
| **Resistance to Change** | **-0.208** | **0.081** | **-2.558** | **0.011** | **-0.208** | **-0.169** |
| ERQ_Reappraisal | -0.145 | 0.173 | -0.836 | 0.403 | -0.145 | -0.046 |
| ERQ_Suppression | -0.376 | 0.200 | -1.877 | 0.061 | -0.376 | -0.098 |
| REIS24_Rational | -0.007 | 0.123 | -0.057 | 0.955 | -0.007 | -0.003 |
| REIS24_Experiential | 0.026 | 0.105 | 0.249 | 0.803 | 0.026 | 0.013 |
|  | | | | | | |
| **COVARIANCES** | | | | | | |
|  | **Estimate** | **Std. Err** | **z value** | **P (>\|z\|)** | **Std. lv** | **Std. all** |
| INQ_Inclusion ~~ | | | | | | |
| **JSI_JobSatisfaction** | **80.569** | **9.611** | **8.383** | **< .001** | **80.569** | **0.503** |
| **CSS_CommunicationSatisfaction** | **85.547** | **9.497** | **9.008** | **< .001** | **85.547** | **0.551** |
| JSI_JobSatisfaction ~~ | | | | | | |
| **CSS_CommunicationSatisfaction** | **76.552** | **9.979** | **7.671** | **< .001** | **76.552** | **0.451** |
|  | | | | | | |
| **VARIANCES** | | | | | | |
|  | **Estimate** | **Std. Err** | **z value** | **P (>\|z\|)** | **Std. lv** | **Std. all** |
| **INQ_Inclusion** | **146.435** | **11.101** | **13.191** | **< .001** | **146.435** | **0.841** |
| **JSI_JobSatisfaction** | **175.177** | **13.280** | **13.191** | **< .001** | **175.177** | **0.845** |
| **CSS_CommunicationSatisfaction** | **164.372** | **12.461** | **13.191** | **< .001** | **164.372** | **0.831** |

| M4 | | | | | | |
| --- | --- | --- | --- | --- | --- | --- |
| **REGRESSIONS** | | | | | | |
|  | **Estimate** | **Std. Err** | **z value** | **P (>\|z\|)** | **Std. lv** | **Std. all** |
| INQ_Inclusion ~ | | | | | | |
| **PQ_Industriousness** | **-0.299** | **0.141** | **-2.117** | **0.034** | **-0.299** | **-0.097** |
| **PQ_Dutifulness** | **0.292** | **0.133** | **2.193** | **0.028** | **0.292** | **0.099** |
| **TOM_Relation** | **0.793** | **0.136** | **5.827** | **< .001** | **0.793** | **0.303** |
| **Resistance to Change** | **-0.215** | **0.058** | **-3.708** | **< .001** | **-0.215** | **-0.186** |
|  | | | | | | |
|  | **Estimate** | **Std. Err** | **z value** | **P (>\|z\|)** | **Std. lv** | **Std. all** |
| JSI_JobSatisfaction ~ | | | | | | |
| **TOM_Relation** | **0.755** | **0.143** | **5.269** | **< .001** | **0.755** | **0.264** |
| **Resistance to Change** | **-0.298** | **0.063** | **-4.712** | **< .001** | **-0.298** | **-0.236** |
|  | | | | | | |
|  | **Estimate** | **Std. Err** | **z value** | **P (>\|z\|)** | **Std. lv** | **Std. all** |
| CSS_CommunicationSatisfaction~ | | | | | | |
| PQ_Industriousness | -0.310 | 0.165 | -1.883 | 0.060 | -0.310 | -0.095 |
| **PQ_Dutifulness** | **0.348** | **0.148** | **2.351** | **0.019** | **0.348** | **0.112** |
| TOM_Target | 0.053 | 0.103 | 0.516 | 0.606 | 0.053 | 0.029 |
| **TOM_Relation** | **0.755** | **0.155** | **4.864** | **< .001** | **0.755** | **0.271** |
| **Resistance to Change** | **-0.242** | **0.069** | **-3.493** | **< .001** | **-0.242** | **-0.196** |
|  | | | | | | |
| **COVARIANCES** | | | | | | |
|  | **Estimate** | **Std. Err** | **z value** | **P (>\|z\|)** | **Std. lv** | **Std. all** |
| INQ_Inclusion ~~ | | | | | | |
| **JSI_JobSatisfaction** | **83.110** | **9.868** | **8.422** | **< .001** | **83.110** | **0.506** |
| **CSS_CommunicationSatisfaction** | **87.907** | **9.747** | **9.019** | **< .001** | **87.907** | **0.552** |
| JSI_JobSatisfaction ~~ | | | | | | |
| **CSS_CommunicationSatisfaction** | **79.266** | **10.296** | **7.699** | **< .001** | **79.266** | **0.453** |
|  | | | | | | |
| **VARIANCES** | | | | | | |
|  | **Estimate** | **Std. Err** | **z value** | **P (>\|z\|)** | **Std. lv** | **Std. all** |
| **INQ_Inclusion** | **149.449** | **11.330** | **13.191** | **< .001** | **149.449** | **0.862** |
| **JSI_JobSatisfaction** | **180.550** | **13.687** | **13.191** | **< .001** | **180.550** | **0.871** |
| **CSS_CommunicationSatisfaction** | **169.510** | **12.851** | **13.191** | **< .001** | **169.510** | **0.863** |

## Machine Learning Classification Method Choice

When considering machine learning classification algorithms, we evaluated different alternative choices. We started our evaluation by plotting the distribution of each predictor and scatterplots of each predictors couple, classified based on the outcome class (low vs. high) (figures available in Supplementary Materials). From a visual inspection of these plots, we found that most outcome variables do not appear to be linearly separable For this reason we a priori excluded all linear classification methods.

Among non-linear algorithms, the methods that are most used in research are k-nearest neighbours (k-NN), deep neural networks, decision trees (DT) and support vector classifiers (SVC; with non-linear kernels). We decided to exclude deep neural networks as we had data from not enough participants. Therefore, we decided to compare the other three methods by testing the 5-fold cross-validation score (CVS) of each model on the training set with default parameters. See the following table for the CVS results.

| METHOD | INQ | JSI | CSS | MEAN | MAX |
| --- | --- | --- | --- | --- | --- |
| *k*-NN | .560 ± .042 | .563 ± .036 | .578 ± .074 | .567 ± .051 | .618 |
| SVC | .560 ± .020 | .563 ± .051 | .595 ± .022 | .573 ± .031 | .604 |
| DT | .558 ± .083 | .549 ± .030 | .569 ± .075 | .559 ± .063 | .622 |

*The table represents the mean ± standard deviation of the 5-fold cross-validation score (CVS) values found using three classification methods (i.e., k-NN, SVC and DT) on the three outcome variables (i.e., INQ, JSI, CSS). The “MEAN” column represents the mean ± average standard deviation values across the three outcome variables. The “MAX” column represents the maximum potential CVS value computed as mean + 1 standard deviation.*

DTs showed the lowest CVS values in all models and were therefore excluded. On the contrary, k-NNs and SVCs with polynomial kernel mostly showed similar CVSs, proving thus to be both suitable methods for our analyses.

In order to further investigate differences between these two methods, we also compared them based on the mean CVS values across the three outcome variables and on the maximum potential CVS value computed as mean + 1 standard deviation. While the greater mean CVS value favoured SVC models, the greater maximum CVS value was reached by k-NNs. Therefore, we obtained further evidence that these two methods are fundamentally equivalent.

We chose to use the k-NN method in our final models as it represents a simpler method to use in datasets with our dimensionality and in an applied context such as predictive HR analytics. Indeed, the higher number of parameters to search for and to be balanced in SVCs makes overfitting avoidance more complex and model computation more expensive.
