# Supplementary figures and images for: Estimating Successful Internal Mobility: A Comparison Between Structural Equation Models and Machine Learning Algorithms
Source: Front Artif Intell. 2022 Mar 25;5:848015. doi: 10.3389/frai.2022.848015 (PMC8990773; doi:10.3389/frai.2022.848015)

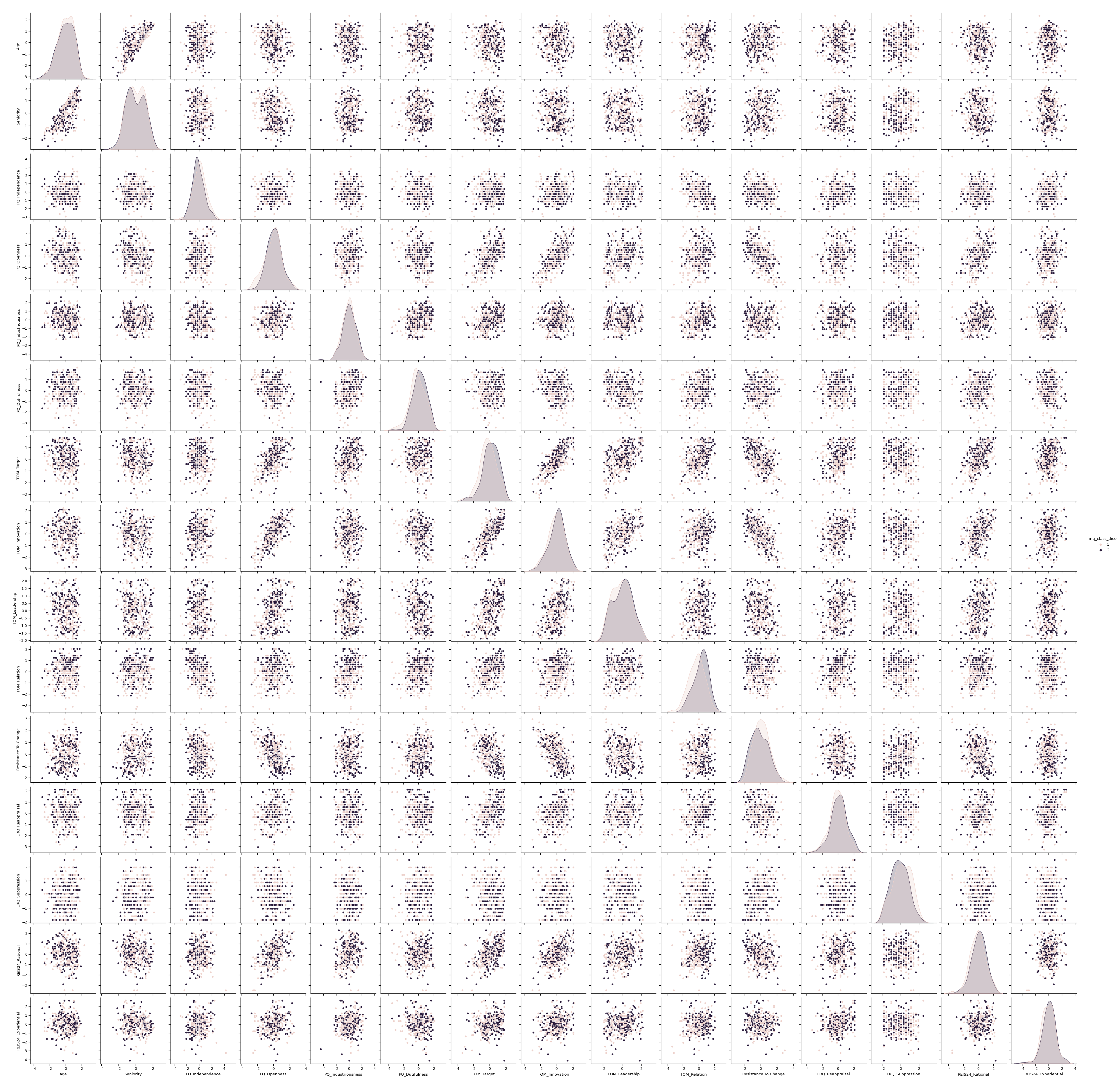

Supplement: Supplementary file 2 [file Image_1.PNG]

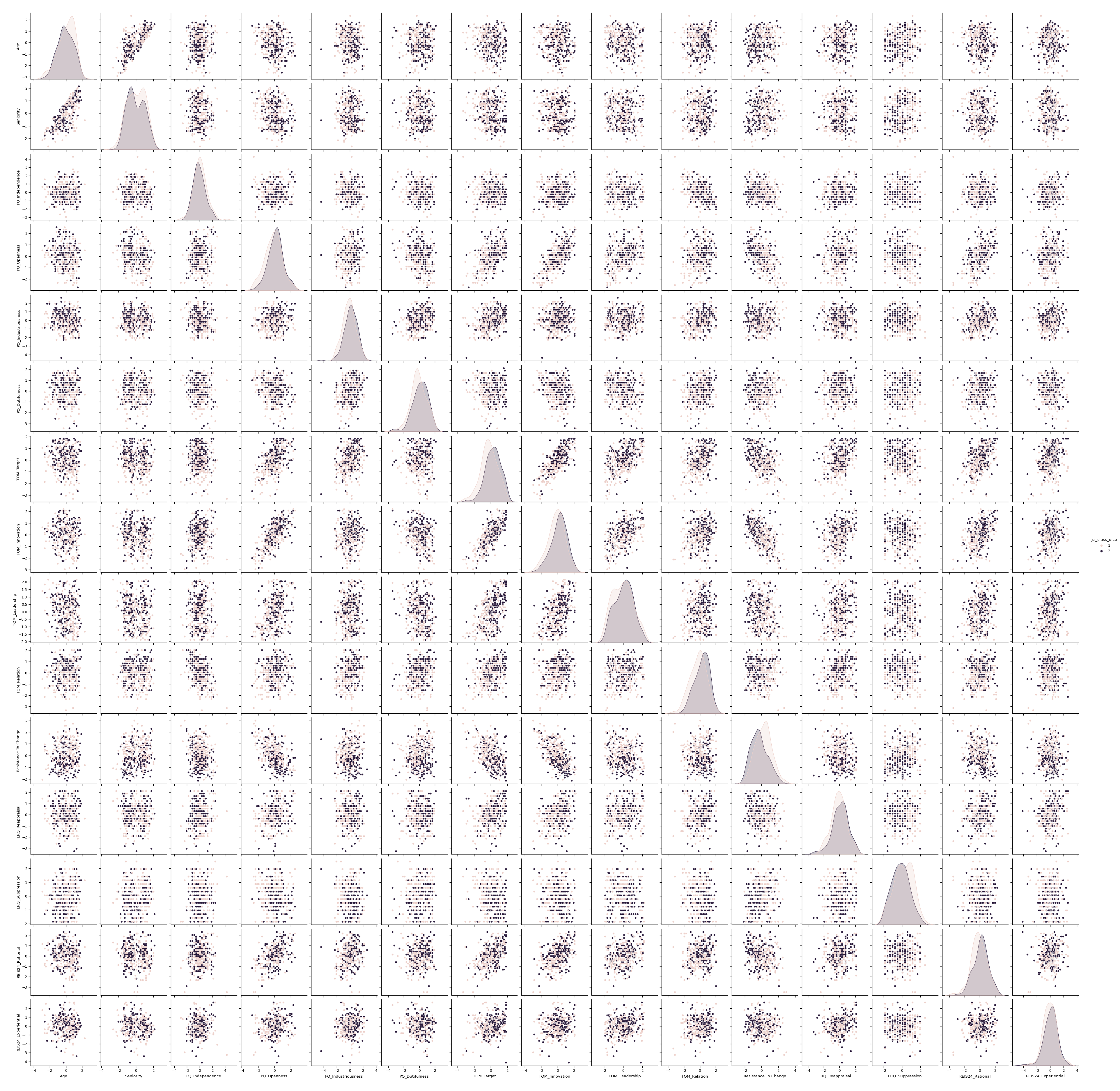

Supplement: Supplementary file 3 [file Image_2.PNG]

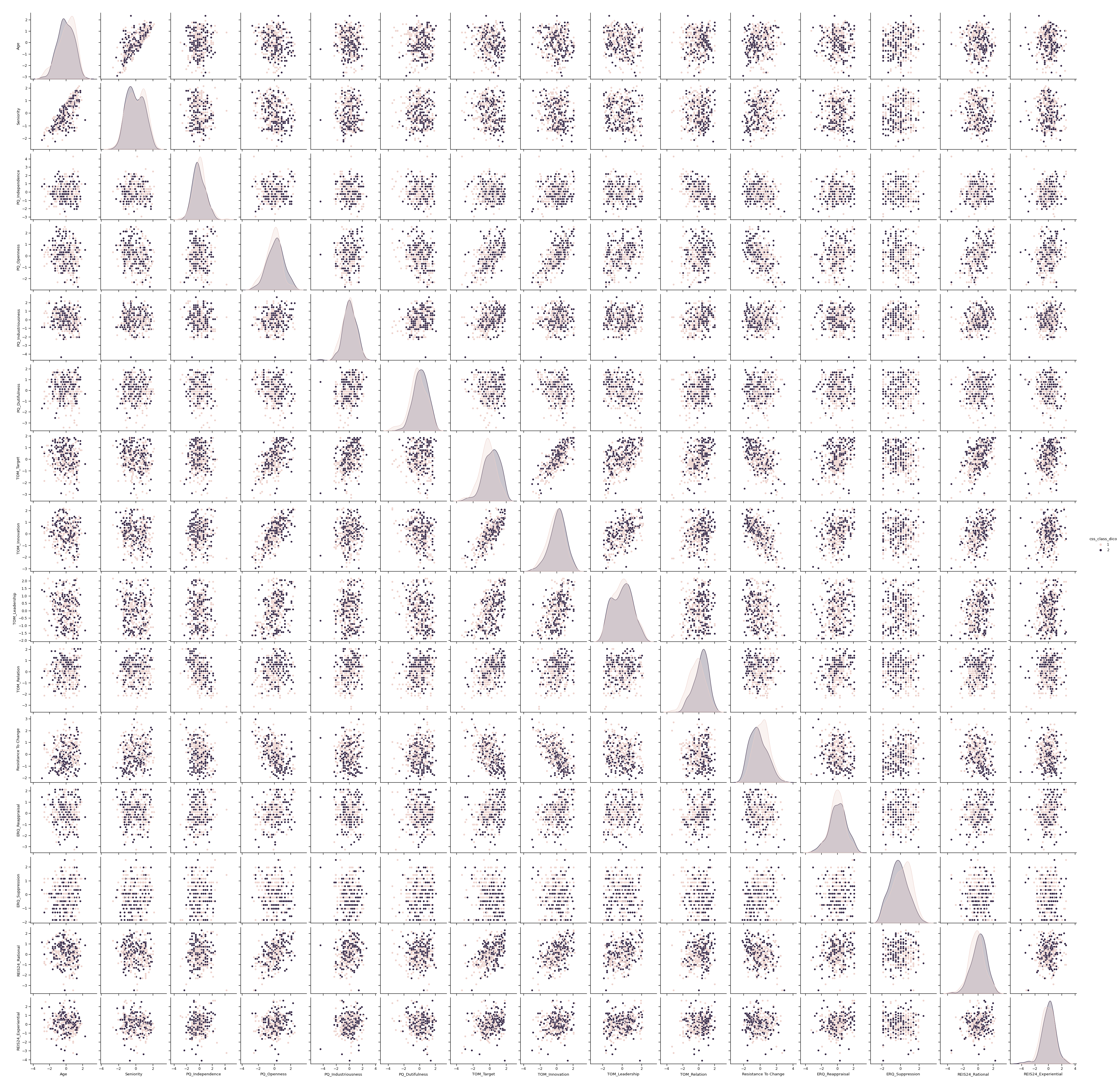

Supplement: Supplementary file 4 [file Image_3.PNG]
